# Personalized neoantigen vaccine prevents postoperative recurrence in hepatocellular carcinoma patients with vascular invasion

**DOI:** 10.1186/s12943-021-01467-8

**Published:** 2021-12-13

**Authors:** Zhixiong Cai, Xiaoping Su, Liman Qiu, Zhenli Li, Xiaolou Li, Xiuqing Dong, Fuqun Wei, Yang Zhou, Liuping Luo, Geng Chen, Hengkai Chen, Yingchao Wang, Yongyi Zeng, Xiaolong Liu

**Affiliations:** 1 The United Innovation of Mengchao Hepatobiliary Technology Key Laboratory of Fujian Province, Mengchao Hepatobiliary Hospital of Fujian Medical University, Xihong Road 312, Fuzhou, 350025 Fujian Province People’s Republic of China; 2 The Liver Center of Fujian Province, Fujian Medical University, Fuzhou, 350025 People’s Republic of China; 3 Mengchao Med-X Center, Fuzhou University, Fuzhou, 350116 People’s Republic of China; 4 Department of Gastroenterology, Second Affiliated Hospital of Wenzhou Medical University, Wenzhou, 325000 People’s Republic of China; 5 School of Basic Medicine, Wenzhou Medical University, Wenzhou, 325000 People’s Republic of China

**Keywords:** Neoantigen vaccine, HCC, Anti-recurrence, Prophylactic treatment, Circulating tumor DNA

## Abstract

**Background:**

Clinically, prophylactic anti-recurrence treatments for hepatocellular carcinoma (HCC) patients after radical surgery are extremely limited. Neoantigen based vaccine can generate robust anti-tumor immune response in several solid tumors but whether it could induce anti-tumor immune response in HCC and serve as a safe and effective prophylactic strategy for preventing postoperative HCC recurrence still remain largely unclear.

**Methods:**

Personalized neoantigen vaccine was designed and immunized for 10 HCC patients with high risk of postoperative recurrence in a prime-boost schedule. The safety and immune response were assessed through adverse events, tissue sequencing, ELISpot, TCR sequencing. The clinical response was evaluated by recurrence-free survival (RFS) and personalized circulating tumor DNA (ctDNA) sequencing.

**Results:**

In the 10 enrolled patients, no obvious adverse events were observed during neoantigen vaccinations. Until the deadline of clinical trial, 8 of 10 patients were confirmed with clinical relapse by imaging, the other 2 patients remained relapse-free. From receiving first neoantigen vaccination, the median RFS of 10 patients were 7.4 months. Among 7 patients received all planned neoantigen vaccinations, 5 of them demonstrated neoantigen-induced T cell responses and have significantly longer RFS after radical surgery than other 5 patients without responsive neoantigens or only with prime vaccination and propensity scores matching control patients (*p* = 0.035). Moreover, tracking personalized neoantigen mutations in ctDNA could provide real-time evaluation of clinical response in HCC patients during neoantigen vaccination and follow up.

**Conclusion:**

Personalized neoantigen vaccine is proved as a safe, feasible and effective strategy for HCC anti-recurrence, and its progression could be sensitively monitored by corresponding neoantigen mutations in ctDNA, and thus provided solid information for individualized medicine in HCC.

**Trial registration:**

This study was registered at Chinese Clinical Trial Registry; Registration number: ChiCTR1900020990.

**Supplementary Information:**

The online version contains supplementary material available at 10.1186/s12943-021-01467-8.

## Introduction

Hepatocellular carcinoma (HCC) is an increasingly serious global pandemic. Especially in China, where new HCC cases account for 55% of the world’s total cases, approximately 422,100 people die from HCC progression per year [1]. Presently, a large number of HCC patients were diagnosed with different degrees of vascular invasion, resulting in low surgical resection rate and poor prognosis. However, previous clinical studies have shown that some HCC patients with vascular invasion in portal venous branch can still benefit from surgery treatment [2, 3], and such patients could be recommended for surgery treatment following the guidelines for Diagnosis and Treatment of Primary Liver Cancer in China (2019 Edition) and EASL Clinical Practice Guidelines [4, 5]. Unfortunately, these patients still suffer a high risk of recurrence and metastasis after surgery. At present, prophylactic anti-recurrence treatments after surgery are very limited clinically. Transcatheter Arterial Chemoembolization (TACE) is the main prophylactic strategy in China, but still lack sufficient supportive evidence while there are no recommended treatments in EASL and NCCN guidelines. Therefore, it is very urgent to develop new and effective strategy for preventing postoperative recurrence.

Neoantigens, epitope peptides solely derived from nonsynonymous mutations of malignant tumor cells, could be present to the cell surface with the major histocompatibility complex (MHC) on tumor cells and thus could be specific recognized by T cells to elicit strong antitumor immune responses. Comparing with tumor-associated antigen based vaccine, neoantigen based cancer therapeutic vaccine has been proved as a promising anti-tumor immunotherapy strategy with maximized therapeutic efficacy and minimized risk of autoimmunity, since such neoantigens are only found in tumor tissues [6]. Neoantigens usually originate from somatic DNA point mutations, RNA editing events, insertion-deletion mutations, gene fusions and so on [7]. Recently, numbers of neoantigen vaccine based clinical trials showed that neoantigens derived from somatic point mutations could induce strong antitumor immune responses in patients with melanoma, small cell lung cancer, as well as gliomas [8–10]. However, whether neoantigen vaccine could induce anti-tumor immune responses in HCC, which tumor mutation burden only ranked in the intermediate position among different types of cancers, with ~ 2.0 mutations/megabase [11, 12], is still in doubt. Previous reports have pointed out that neoantigen burden in HCC patients could reflect tumor evolution during HCC progression and associated with patient’s prognosis, suggesting neoantigens might serve as ideal immunotherapeutic targets for HCC immunotherapy [13, 14]. In addition, RNA editing events have been reported with capability to produce neoantigens [15], providing complementary targets for patients with low mutation burden. However, whether it could be used as potential therapeutic targets to induce robust anti-tumor immune responses in HCC patients also needs to be explored.

Furthermore, real-time and accurate evaluation of treatment efficacy and prognosis is still a great challenge in HCC surveillance. Due to the limited sensitivity and specificity, imaging (CT/MRI) and serum protein biomarkers fail to acutely provide real-time evaluation of tumor burden, especially in monitoring minimal residual disease (MRD) after radical operation. Our previous study has indicated that circulating tumor DNA (ctDNA), a short DNA fragment derived from tumor cell in plasma, could function as noninvasive and sensitive biomarker for monitoring real-time HCC burden by tracking personalized tumor mutations [16]. Jia et al. also indicated that by tracking personalized neoantigen sites in ctDNA could accurately reflect the clinical response during checkpoint blockade immunotherapy in non-small cell lung cancer [17]. Thus, ctDNA might provide a potential strategy to sensitively and accurately assess clinical response during neoantigen based immunotherapy and should be thoroughly investigated.

In this prospective clinical study, we enrolled 10 patients with resectable HCC and vascular invasion in portal venous branch, which indicated high risk of recurrence after radical operation. Following personalized neoantigen identification for each patient, we comprehensively evaluated whether personalized neoantigen long peptide vaccine could serve as a safe and effective strategy for preventing postoperative recurrence. Furthermore, we designed personalized somatic mutation panel, including neoantigen mutation sites, for ctDNA sequencing to monitor the clinical response during neoantigen based immunotherapy.

## Methods

### Clinical trial design and treatment

This study was investigator initiated, single-arm, open-label clinical trial at Mengchao Hepatobiliary Hospital of Fujian Medical University in China and was registered at Chinese Clinical Trial Registry (http://www.chictr.org.cn/; ChiCTR1900020990). This trial was aimed to study the safety and feasibility of personalized neoantigen vaccine for HCC anti-recurrence after hepatectomy. The corresponding designment, protocol and amendment were all approved by the Institution Review Board of Mengchao Hepatobiliary Hospital of Fujian Medical University (Fujian, China). All treatments and sample collections in this trial were in accordance with Declaration of Helsinki and the International Conference on Harmonization Guidelines for Good Clinical Practice. The informed written consents were signed from all enrolled patients. The safety and feasibility of neoantigen vaccine were the primary endpoints and the corresponding immune response and relapse free progression (RFS) were the secondary endpoints. The key inclusion criteria of enrolled patient is seen below: (1) aged 18 to 75 years old male and female, with serum bilirubin not higher than 1.5× upper limit of normal (ULN) and ALT or AST not higher than 2.0× ULN; (2) be diagnosed with resectable HCC or intrahepatic cholangiocarcinoma(CC) without any metastasis; (3) the existence of tumor thrombus in portal venous branch should be confirmed by histopathology or visible to the naked eye during the surgery. Key exclusion criteria included: (1) Patients with HIV infection, HCV infection, serious coronary artery disease or other diseases that the researchers consider not suitable to be included in this study; (2) Patients with history of bone marrow transplantation or organ transplantation; (3) Patients with any form of immunodeficiency or history of autoimmune disease; (4) Patients received prior treatment with any other immunotherapy within 1 month or have fewer than five identified actionable neoepitopes.

After diagnosed with HCC, all enrolled patients firstly received radical surgical resection and then underwent prophylactic TACE within 2 months. Subsequently, personalized neoantigen vaccines were produced and administered subcutaneously at a prime-boost schedule. When the patient occurs HCC recurrence confirmed by imaging, he will reach the end of the clinical trial and receive standard clinical treatment. Physical examination, routine blood and biochemical tests and electrocardiogram monitoring were used to assess the safety of each neoantigen vaccination.

Meanwhile, we included 20 HCC patients who did not receive neoantigen vaccine as patient-controls though propensity scores matching (PSM). All PSM-control patients have similar clinical parameters with those 10 enrolled patients, such as clinical treatment (laparoscopy plus prophylactic TACE treatment), sex, age, vascular invasion, hemoglobin level (≥100 g/dl) and platelet level(≥80/l). Individuals were subsequently matched using a 1:2 nearest-neighbor matching algorithm within a caliper of 0.2 in PSM. All potential confounding factors in Supplementary Table S1 as covariates.

HCC and peritumor tissues were collected at the time of hepatectomy. Serial blood samples, including plasma and peripheral blood mononuclear cells (PBMCs), were collected during whole clinical course, including preoperative, postoperative, neoantigen vaccination time points and follow-up time points. Clinical response assessment (including imaging examination, ctDNA sequencing) and immune responses (including tissue sequencing, immunohistochemical staining, ELISpot, TCR sequencing, peripheral blood T lymphocyte subsets and cytokine assay) were conducted during neoantigen vaccination and follow up. Those detailed methods were shown in Supplementary Method.

### Tissue sequencing and epitope prediction

To identify potential neoantigen profiling, HCC and matched peritumor tissues were used for whole exome sequencing with coverage depths of 200× and transcriptomic sequencing. Briefly, after DNA/RNA extraction from HCC and matched peritumor tissue of each patient, exome and transcriptome libraries were further constructed according to the manufacturer’s instructions, respectively. Then those libraries were sequenced by Fulgent. Co., Ltd. on Illumina Novaseq 6000 platform (paired end, 150 bp).

The four-digit genotype of classical HLA class I genes (HLA-A, HLA-B and HLA-C) were assessed by OptiType [18] based on RNA-seq data, while HLA class II alleles (HLA-DRB1) were assessed by Seq2HLA. Somatic mutations were called using exome sequencing data by Mutect2 and Somaticsniper, and consistent somatic mutations were further confirmed by DeepSNV. All the somatic mutations were further validated at RNA level and somatic mutations with > = 20× depth and variant allele frequency (VAF) > = 0.05 in tumor RNA-seq data were kept. Immunogenicity of all remaining mutations were then evaluated using pVAC-Seq pipeline [19]. For each missense mutation, the binding affinity of 8 ~ 11 mer peptide containing mutated amino acids to patient’ s HLA class I alleles were predicted by NetMHCpan, NetMHC NetMHCcons, PickPocket, MHCflurry, while the binding affinity of corresponding 15-mer peptide to HLA class II alleles was predicted by NetMHCpanII. Mutations that produce mutant peptides with median IC50 < 500 nM of HLA class I or IC50 < 500 nM HLA class II alleles were considered as predicted neoantigens. All the predicted neoantigens were adopted to prepare long peptides (27mer) if the number was less than 30. If predicted neoantigens greater than 30, epitopes were chosen in priority with the following rank order: (1) with both strong binding affinity (IC50 < 150 nM) to HLA class I and HLA class II alleles; (2) with either strong binding affinity to HLA class I and HLA class II alleles; (3) with higher VAF at RNA level; (4) with modest binding affinity (150 < IC50≦500) to both HLA class I or HLA class II alleles.

### Personalized neoantigen long-peptide vaccine synthesis and vaccination

To generate personalized neoantigen vaccines, up to 30 potential neoantigen mutations were selected and prioritized for clinical-grade long peptides synthesis (27 amino acids in length) by the standard solid-phase synthetic peptide chemistry with GMP-like standard (> 98% purity, endotoxin concentration was less than 0.01 EU/g). Among enrolled patients, 6~20 of neoantigen peptides were successfully synthesized in time for neoantigen vaccine preparation. All such peptides were grouped into 2 ~ 4 pools (designated as pools 1 ~ 4, each pool contained 3 ~ 5 peptides, 0.3 mg/peptide) and received strict quality control, including sterility test, pyrogen detection and abnormal toxicity test. Then neoantigen vaccine pools were further mixed with 0.5 mg poly:IC (polyinosinic-polycytidylic acid injection, Guangdong South China Pharmaceutical. Co) as adjuvant and then subcutaneously (s.c.) injected at bilateral underarm and groin area, circularly. For neoantigen vaccination, patients were received neoantigen vaccine on days 1, 4, 8, 15 and 22 as the prime phase and on days 90 and 140 as the boost phase. Vaccination time is allowed within 15 days of the scheduled vaccination time in the boost phase.

### Statistical analysis

Data from all enrolled patients received at least 5 planned prime phase vaccinations were included in the safety and clinical outcome evaluation. Descriptive statistics was used to determine safety of neoantigen vaccine. The RFS curves, immune response curves and ctDNA dynamic curves were plotted with GraphPad Prism 6 or R software.

## Results

### Study design and patient characteristics

**Fig. 1:**
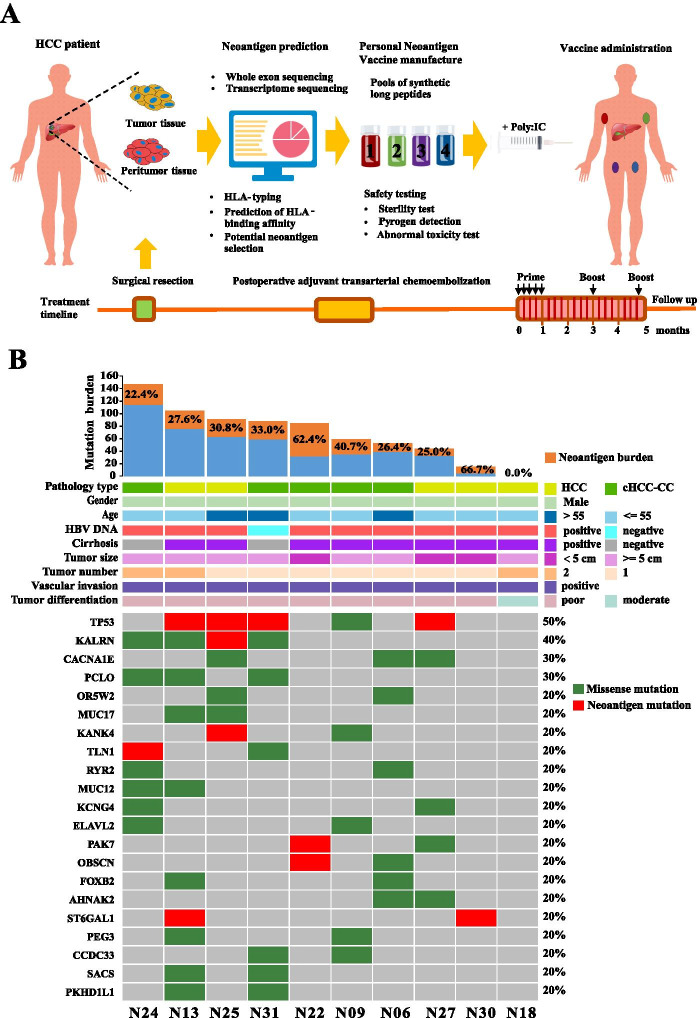
The overview of study design, patient characteristics, and neoantigen profiles. **A** The infusion program of personalized neoantigen vaccine for HCC patients with vascular invasion. **B** Number of somatic mutations and neoantigens detected in each patient’s HCC tissue and corresponding clinicopathologic information in 10 enrolled patients. The percentage shows the proportion of the number of neoantigen mutations to the number of total somatic mutations. Heatmap summarized the somatic/neoantigenic hotspot gene profile in enrolled HCC patients. The neoantigen mutations of patient N06 and N09 all come from personalized gene mutations rather than hot gene mutations

In this single-arm prospective clinical study, personalized neoantigen profiling was firstly performed for each newly diagnosed HCC patient with vascular invasion based on whole exome sequencing data and transcriptomic sequencing data of surgically resected tumor tissue and matched peritumor tissue (Fig. 1A). For each patient, 6 ~ 20 personalized neoantigen long peptides (27 aa) derived from somatic point mutations or RNA editing events were synthesized for vaccine manufacture and were divided into 1~4 pools (termed as pools 1~4, each pool contained 3~5 long peptides). Following strict quality control, the neoantigen vaccines together with poly:IC were administered at bilateral underarm and groin of HCC patient in a prime-boost schedule (days 1, 4, 8, 15 and 22 as the prime phase, days 90 and 140 as the boost phase) after radical surgery and prophylactic TACE.

According to the investigator’s assessment, 10 patients with resectable HCC were included for evaluating the safety and efficacy of neoantigen vaccines. As shown in Fig. 1B, among 10 enrolled patients, pathological diagnosis confirmed that 5 of them were HCC and other 5 patients were diagnosed as combined HCC and intrahepatic cholangiocarcinoma (cHCC-CC). Nine patients diagnosed with HBV infection and 8 of them had concomitant liver cirrhosis. The average tumor diameter of 10 patients was 7.7 cm (range, 2.8~11 cm).

In total, 780 nonsynonymous somatic single-nucleotide variants (SNVs) were identified, with an average of 78 mutations (range, 7~148) in each patient using whole-exome sequencing of tumor tissue and matched peritumor tissue (Fig. 1B). Meanwhile, 33.5% (range, 0~66.7%) of these somatic mutations were further confirmed for expression by transcriptomic sequencing analysis (variant allele frequency > = 0.05) and identified as neoantigens by epitope prediction simultaneously (IC50 < 500 nM for HLA class I or II binding). The detailed HLA type and neoantigen sequence for neoantigen vaccine preparation of each enrolled patients were shown in Supplementary Table S2.

As shown in Fig. 1B, HCC frequently missense mutated genes in protein coding region, such as TP53 and KALRN were observed in 50% and 40% of enrolled patients, respectively. As expected, among all identified neoantigens, neoantigens originated from TP53 mutations were observed in 40% (4/10) patients. Noteworthily, in one enrolled patient N18, no nonsynonymous somatic SNVs were identified in DNA level. Fortunately, 20 tumor specific nonsynonymous RNA editing sites were identified and 6 of them were identified as neoantigens (Supplementary Fig. S1). Presently, the immunogenicity of neoantigen derived from RNA editing sites has been proved to have potential immunogenicity [15]. After fully informing potential treatment benefits and risks and obtaining informed consent from patient N18, we prepared a personalized neoantigen vaccine based on RNA editing sites for patient vaccination in a prime-boost schedule.

### The safety of neoantigen vaccination

The median duration from hepatectomy to first vaccination was 86 days, ranging from 59 to 159 days. All 10 enrolled patients received 5 planned prime phase vaccinations and 7 of them underwent another 2 boost vaccinations. No obvious treatment related adverse events were observed and routine blood/biochemical tests also showed no obvious abnormalities during vaccinations (Supplementary Tables S3 and S4). Only some minor side effects (Grade 1), such as injection site reaction and fatigue, were reported after the vaccine injection but quickly disappeared without additional treatments. These results supported the high safety of neoantigen vaccines.

### Clinical outcome and immune response monitoring during vaccination and follow up

**Fig. 2:**
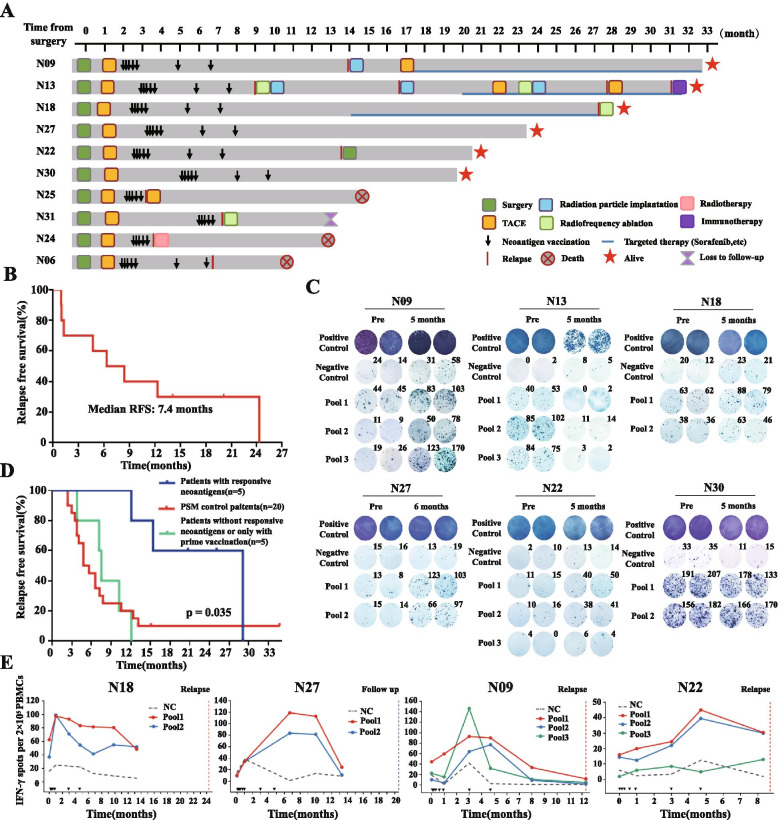
Clinical outcome and immune response monitoring in enrolled HCC patients during vaccination and follow up. **A** The detailed timeline presentation of clinical treatments, vaccinations and clinical outcomes for 10 enrolled HCC patients from surgery treatment until the deadline of clinical trial. **B** The Kaplan-Meier survival curve of RFS after receiving first neoantigen vaccination in enrolled 10 patients. **C** The Ex vivo IFN-γ ELISpot responses for PBMCs stimulated by personalized neoantigen pools between pre- and post-neoantigen vaccinations in 6 patients. **D** The Kaplan-Meier survival curve of RFS after radical surgery among patients with responsive neoantigens, patients without responsive neoantigens or only with prime vaccination and propensity scores matching control patients. **E** Long-term monitoring of Ex vivo IFN-γ ELISpot response for PBMCs stimulated by personalized neoantigen pools in 4 patients. PC indicated as positive control and NC indicated as negative control

The detailed timeline presentation of clinical treatments, vaccinations and clinical outcomes were shown in Fig. 2A. From November 26, 2018 to June 30, 2021, the median follow-up time of these 10 enrolled patients was 20.1 months (range, 10.9~32.7 months). Eight out of ten patients were confirmed with clinical relapse by MRI/CT imaging, with the median relapsed-free survival (RFS) of 8.3 months after radical surgery; the other 2 patients remained relapsed-free (mean follow-up time of 21.4 months). Kaplan-Meier survival analysis showed that the median RFS of enrolled patients after receiving first neoantigen vaccination was 7.4 months (Fig. 2B).

Generally, neoantigen vaccines need to be fully administered by primer and boost vaccinations, and take 2 ~ 5 months to build up robust neoantigen-specific anti-tumor immune response in vivo. However, 3 patients (N24, N25 and N31) were relapsed within 2 weeks after prime phase vaccination and thus not received boost vaccinations (Fig. 2A), which might leave insufficient time to induce enough anti-tumor immune response. Therefore, in vitro IFN-γ ELISpot assay was performed to monitor immune response of other 7 patients who received all boost vaccines by using autologous PBMCs stimulated by neoantigen pool-pulsed autologous DC cells. As shown in Supplementary Fig. S2, patient N06 did not generate obviously IFN-γ responses during vaccinations. However, in 3 patients (N09, N22 and N27), the neoantigen specific reactivity of PBMCs against neoantigen peptide pools corresponding to neoantigen vaccines was considerably generated after all vaccinations when compared with corresponding negative control and pre-vaccination sample (Fig. 2C). Meanwhile, early PBMC reactivity against neoantigen peptide pools before vaccination was observed in 3 patients (N13, N18 and N30), which may probably due to releasing tumor neoantigens of MRD by the prophylactic TACE treatment to further activate specific T cells. However, during vaccinations, we also found that the neoantigen immune response of patient N13 decayed rapidly, and could not detect after boost vaccination (Supplementary Fig. S3). This mean that neoantigen vaccine could not successfully activate the neoantigen-induced T cell responses in patient N13. In contrary, after all vaccinations, the neoantigen specific reactivity in patient N18 was considerably stronger than pre-vaccination; patient N30 maintained a similar immune response intensity as before vaccination, which may be induced by neoantigen vaccines and prophylactic TACE, or that patient’s immune system was sensitive to neoantigens. Meanwhile, the neoantigen specific reactivity of PBMCs against each neoantigen individually was further confirmed that 36 out of 51(70.6%) individual long peptides could significantly induced measurable peptide-specific immune response in 5 patients (N09, N18, N22, N27and N30) and other 2 patents (N06 and N13) did not have responsive neoantigens after completed neoantigen vaccination (Supplementary Fig. S4). Interestingly, we found that the 6 neoantigen peptides derived from RNA editing in N18 patients showed 100% immune response rate (Supplementary Fig. S4), suggesting RNA editing sites could indeed serve as neoantigen candidates in patients with low tumor mutation burden. Accordingly, 5 patients (N09, N18, N22, N27 and N30) were identified with responsive neoantigens after vaccinations. Analysis combining clinical data further indicated that patients with responsive neoantigens after fully vaccinations had significantly longer RFS after radical surgery than those without responsive neoantigens or only with prime vaccination and propensity scores matching control patients enrolled in study (median RFS: 19.3 vs 6.7 vs 4.8 months, *P* = 0.035, Fig. 2D). Meanwhile, we also investigated the dynamic change in proportions of peripheral blood T lymphocyte subsets and serum levels of 6 cytokines (IL-2, IL-4, IL-6, IL-10, TNF-α and IFN-γ) in 10 enrolled patients during neoantigen vaccinations (Supplementary Table S5), but did not find a significant correlation with patients’ neoantigen response or RFS (data no shown). These results indicated that neoantigen-induced specific IFN-γ response can be used as an indicator for the effectiveness of neoantigen vaccine.

Then, we further evaluated the durability of the neoantigen-induced immune response in 4 patients (N09, N18, N22 and N27) who had follow-up PBMC samples after neoantigen vaccination. As shown in Fig. 2E, during follow up, neoantigen-specific immune response was still maintained relative strong at the 10 month after vaccination in 2 patients (N18: average 67.8 spots in 2 neoantigen pools; N27: average 97.3 spots in 2 neoantigen pools), but already relatively week at the 8 month after vaccination in patient N09 (average 17.8 spots in 3 neoantigen pools) and patient N22 (average 24.5 spots in 3 neoantigen pools). Accordingly, the RFS of N18 and N27 patients is indeed longer than that of N09 and N22 patients (Fig. 2A). Overall, these results in our cohort suggested that personalized neoantigen vaccine could serve as an effective strategy to induce robust immune response in HCC patients.

### Immune microenvironment dynamics after neoantigen vaccination

**Fig. 3:**
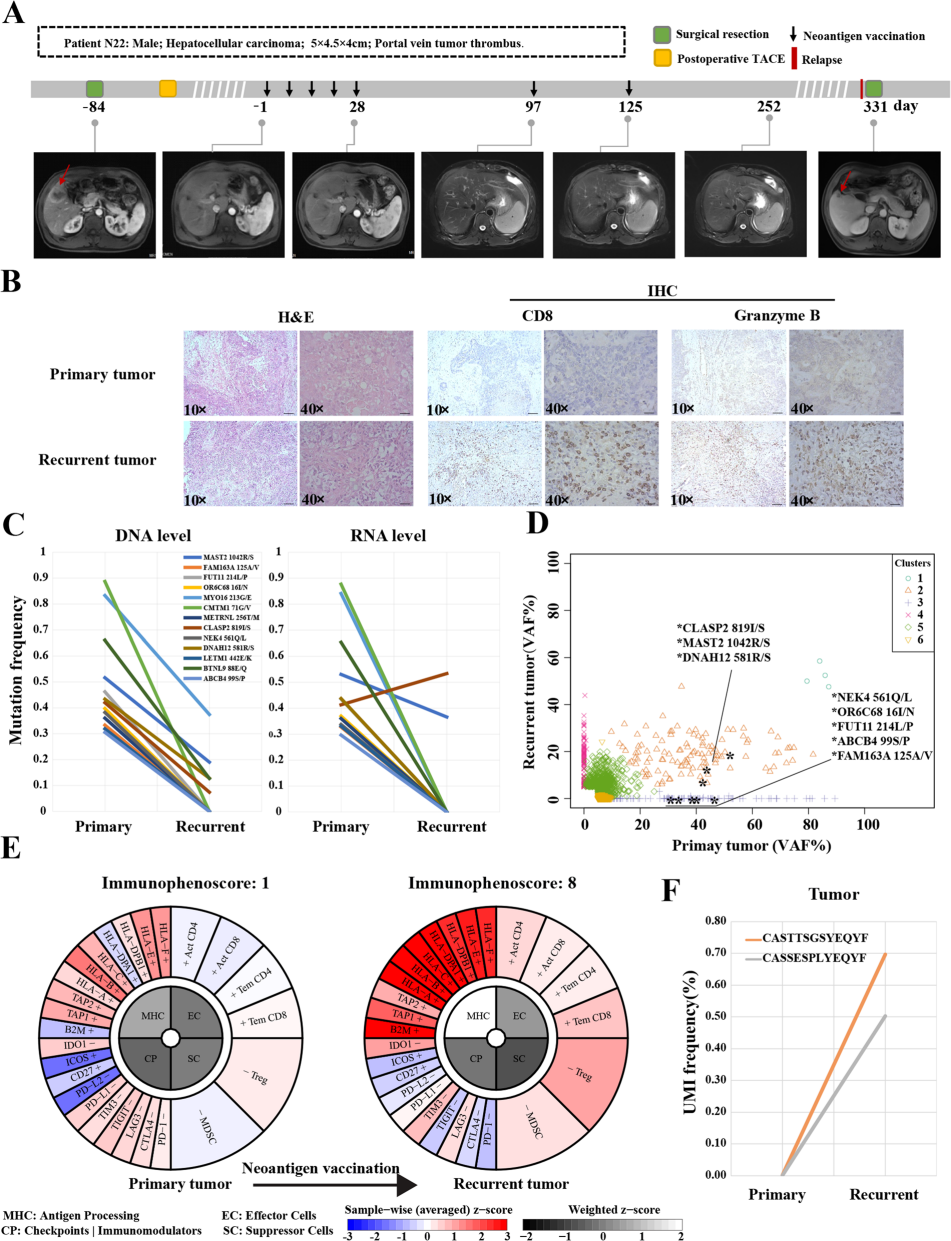
Clinical response and immune microenvironment dynamics for HCC patients during neoantigen vaccination and follow up. **A** Clinical event timeline and corresponding imaging of patient N22. Take the first dose of neoantigen vaccine as day 1. **B** Hematoxylin-eosin staining and Immunohistochemical staining of CD8 and granzyme B in primary tumor and recurrent tumor. **C** The mutation allele frequencies of treated neoantigen mutations in DNA level and RNA level between primary tumor and recurrent tumor. **D** The clonal structure dynamics between primary tumor and recurrent tumor. The asterisk indicated as neoantigen mutation. **E** Immunophenoscore diagrams between primary tumor and recurrent tumor. **F** TCR clone dynamics between primary tumor and recurrent tumor. VAF: variant allele frequency

To explore the dynamics of immune microenvironment during neoantigen vaccination, we performed a toughly evaluation of patient N22 who had recurrent tumor tissue collected after neoantigen vaccination treatment. Patient N22 is a 46-year-old male, firstly diagnosed with HBV related HCC (MRI scan: 5.0 × 4.5 × 4.0 cm, at segment 5 of liver) at Mengchao Hepatobiliary Hospital of Fujian Medical University. Then this patient received surgical resection and underwent prophylactic TACE after 1 month. Pathological examination showed that his tumor was an invasive cHCC-CC with stem cell characteristics. His local lesions showed visible vascular tumor thrombi and some circulating tumor cells, indicating high risk of recurrence after radical operation. Sequencing data presented 44 potential neoantigens from 85 nonsynonymous somatic mutations and 13 of them were successfully synthesized in time and used for personalized neoantigen vaccine preparation. Three months after surgery, he started to receive neoantigen vaccination in planned prime-boost schedule (Fig. 3A). One years after surgical operation, MRI scan revealed a 1.4 cm recurrent lesion in primary HCC origin and he was then treated with second hepatectomy. Immunohistochemical staining revealed that the infiltration of CD8 positive T cells and granzyme B secretion in recurrent tumor tissues were obviously increased compared with the primary tumor (Fig. 3B). The recurrent tumor was further subjected to whole-exome sequencing and transcriptomic sequencing to assess the influence of neoantigen vaccination on tumor immune microenvironment. Analysis of sequencing data showed that the mean mutation allele frequencies for 13 neoantigen mutations identified in primary HCC were both obviously decreased by 89% at DNA level and 85% at RNA level in recurrent tumor, respectively (Fig. 3C); while the mean frequencies of other 72 somatic mutations in recurrent tumor were also correspondingly declined (72% in DNA level and 67% in RNA level, Supplementary Fig. S5). Meanwhile, analysis of clonal structure dynamics between primary tumor and recurrent tumor revealed that 8 neoantigen mutations (other 5 neoantigen mutations were excluded due to copy number variation) are mainly located in cluster 2 and 3, which are significantly shrunk in recurrent tumors; and new clone (cluster 4) was indeed identified in recurrent tumors, suggesting tumor evolved under the pressure of neoantigen based immunotherapy to achieve tumor immune escape (Fig. 3D). Meanwhile, 9 new neoantigen mutations were also found in recurrent tumor (Supplementary Table S6). These results suggested that the proportion of tumor cells carrying these neoantigens in recurrent tumor significantly shrunk under neoantigen based immunotherapy. Furthermore, the heatmap analysis of immune cell infiltration by transcriptomic data showed that immune effector cells were significantly accumulated in recurrent tumor including activated CD4+ T cells, activated CD8+ T cells, natural killer cells, and immature dendritic cells (Supplementary Fig. S6). Immunophenoscore diagrams between primary HCC and recurrent tumor also indicated that the immune microenvironment in the recurrent tumor turn into “hot” with enhanced antigen presentation ability through MHC molecules, increased number of effector T cells, decreased expression of immune checkpoints in T cells (Fig. 3E). Moreover, we performed T cell receptor (TCR) sequencing in primary and recurrent tumor tissues to identify potential neoantigen associated TCR clone after vaccination. Two new TCR clones (CASSESPLYEQYF and CASTTSGSYEQYF) were detected in recurrent tumor, suggesting the presence of neoantigen-induced specific T cells (Fig. 3F). Overall, these results indicated that the neoantigen vaccine successfully activated the anti-tumor activity of T cells and could invade into the tumor site.

### The performance of ctDNA for evaluation of immune and clinical responses

**Fig. 4:**
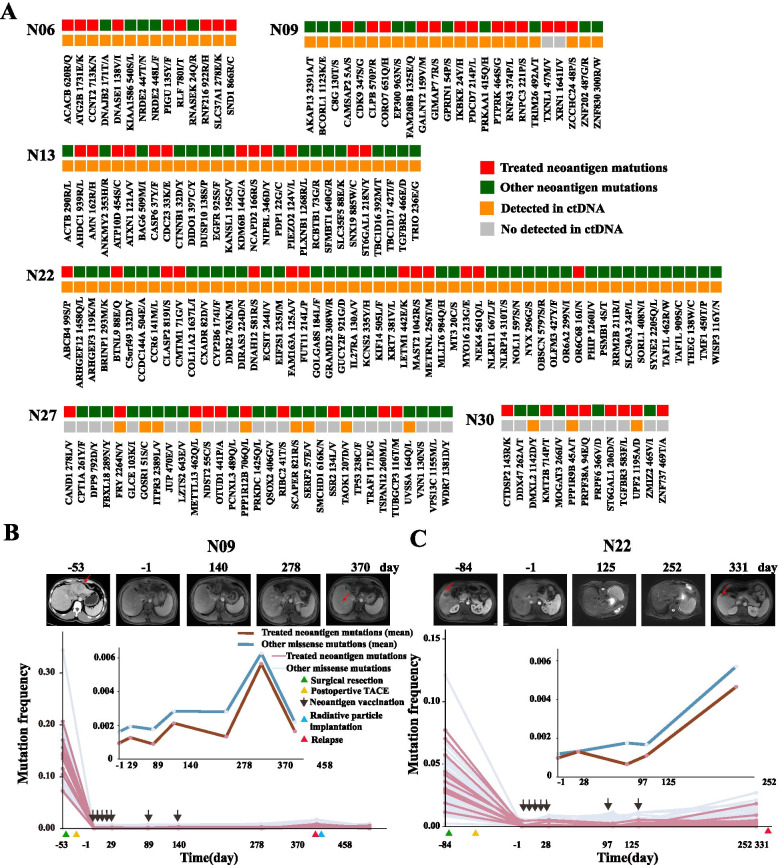
Tracking personalized nonsynonymous somatic mutations in ctDNA during the whole clinical course. **A** The profiling of neoantigen mutations and other somatic mutations detected in HCC and matched preoperative plasma. The time-course demonstration of quantified levels of treated neoantigen mutations and other somatic mutations in patient N09 (**B**) and N22 (**C**), respectively. The small picture displays the dynamic of mean mutation allele frequencies in treated neoantigen mutations and other somatic mutations during neoantigen vaccination and follow up

Real-time monitoring the immune and clinical responses is vital for assessing the responsiveness of neoantigen vaccination and predicting its efficacy. In this study, we monitored the clinical response of 6 enrolled patients with fully neoantigen vaccinations (except for N18 since his neoantigen derived from RNA editing) by tracking the dynamics of their personalized nonsynonymous somatic mutations in ctDNA. As shown in Fig. 4A and Supplementary Fig. S7, an average of 74 and 78.9% positive rate for neoantigen sites (15.4~100%) and other somatic mutations (29~100%) were observed in preoperative plasma. However, in patient N27 and N30, most neoantigen and other mutation sites were not well detected at ctDNA levels, resulting in inability to track tumor burden during clinical course. In other 4 patients, the dynamic change of neoantigens and other mutations in ctDNA were both well consistent with tumor burden evaluated by CT/MRI images during neoantigen vaccination and follow up (Fig. 4B-C and Supplementary Fig. S8A-B).

Meanwhile, we also found postoperative ctDNA remained at a detectable level for those 4 patients before neoantigen vaccination, suggesting the existence of MRD even after radical surgery and following prophylactic TACE. The detailed ctDNA frequency of neoantigen mutations and other somatic mutations in each patient was shown in Supplementary Table S7. In 2 patients identified with neoantigen responses (N09 and N22), the mean frequency of overall ctDNA (treated neoantigen mutations and other mutations) was rising during first month of receiving five prime vaccinations, suggesting that MRD is progressing (Fig. 4B-C). More interestingly, we then observed different ctDNA dynamic patterns of these 2 patients in the following plasma. From day 30 to day 90 during neoantigen vaccination, patient N09 showed both decrease in the mean frequency of treated neoantigen ctDNA and other ctDNA; while in N22 patients, only treated neoantigen ctDNA showed an obviously decline and the frequency of other mutations in ctDNA is still slowly rising. After that, the frequency of overall ctDNA in patient N09 were increased from day 90 to day 140 but then decreased from day 140 to day 278 after receiving 2 booster vaccines; afterwards, his overall ctDNA frequency was increased again until recurrence happening. However, in the similar period, the overall ctDNA frequency of patient N22 was continuously increasing even after receiving the two booster vaccines until recurrence happening at the day 331. This might be due to that the immune response induced by the neoantigen vaccine was insufficient to inhibit tumor progression in the boost phase or the HCC tumor occurred immune escape under the pressure of neoantigen immunotherapy. Significantly, the mean frequency dynamics of neoantigen mutations and other mutations observed in series of patient N22 ctDNA samples was well consistent with the results found in recurrent tumor, that is, the mean mutation frequency of other mutations is higher than that of treated neoantigens, implying that the immune response induced by neoantigen vaccine preferentially killed tumor cells carrying those neoantigens while other cells gained growth superiority. In other two patients (N13 and N06) without obvious neoantigen response detected in PBMCs after fully vaccinations, the overall ctDNA frequency did not show a similar pattern like in N09 or N22 (Supplementary Fig. S8). In summary, these results proved that real-time evaluation of the immune and clinical responses in HCC could be achieved by tracking personalized neoantigen mutations in ctDNA during neoantigen vaccination.

## Discussion

The high recurrence rate of HCC after radical surgery is the main reason for poor prognosis of patients. In China, the prophylactic TACE is the main anti-recurrence strategy for HCC but remains with controversies. Some clinical studies have shown that HCC patients with PVTT receiving prophylactic TACE after radical surgery could effectively prolong the recurrence-free survival time of about 2.4 months when compared with those without prophylactic TACE treatment, but other studies indicated that this treatment had poor anti-recurrence effect and considerable toxicity [20–23]. Therefore, it is extremely necessary to develop novel effective and safe anti-recurrence strategies. Neoantigen vaccines, due to their high specificity, low side effects, and easy preparation, can be used as a potential strategy for anti-recurrence in solid tumors. More importantly, since sufficient tumor tissue and para-cancerous tissue can be likely obtained through surgery, we can comprehensively and accurately identify personalized neoantigen profiling by high-throughput sequencing; meanwhile, postoperative patients are not as prone to rapidly disease progression as terminal patients, sufficient time and resource are available for the preparation of neoantigen vaccines. In this clinical study, 10 enrolled patients successfully received personalized neoantigen vaccination with no obvious adverse events after radical surgery and prophylactic TACE. Their median RFS time reached 11.3 months after surgery. Interestingly, we also found that 5 patients who successfully induced a strong neoantigen response after fully vaccination had longer RFS than those without responsive neoantigens or only with prime vaccinations and PSM control patients. Although, due to the small sample size, it is necessary to further expand the sample size for verification. Therefore, neoantigen vaccination might be a potential strategy for developing anti-recurrence treatments in HCC.

In addition, real-time monitoring the clinical response of neoantigen vaccination is crucial to provide essential assistance for doctors’ clinical decisions. Due to the shortcomings of sensitivity and specificity, clinical protein markers or imaging scans could not effectively monitor the clinical response of neoantigen based immunotherapy in patients with invisible tumor burden. In this clinical trial, we firstly try to real-time monitor treatment outcome of neoantigen vaccination by tracking the patient’s personalized neoantigen mutations and other mutations in plasma ctDNA. Significantly, we observed two different dynamic patterns of neoantigens and other mutations in ctDNA: one is that the dynamic change pattern of neoantigen mutations is similar with that of other mutations during neoantigen vaccinations, implying that patient may have low tumor heterogeneity, that is, most tumor cells contain certain neoantigen mutations and the immune response induced by neoantigen vaccination could specifically kill tumor cells carrying these neoantigens. In such situation, the neoantigen mutations and other mutations in ctDNA might undergo similar changes. Another is that the dynamic change pattern of neoantigen mutations is not consistent with the patterns of other mutations, suggesting that patient may have high tumor heterogeneity, and only a part of the tumor cells contain neoantigen mutations. These results gave solid evidence for judging clinical response and maintenance time of neoantigen based immunotherapy, which would in-time help doctors to adjust therapeutic strategy.

Meanwhile, based on the results of ctDNA, we also found that the duration of anti-tumor immune response induced by neoantigen vaccination is still limited, which is not enough to completely inhibit MRD progression. There are two possible reasons: on the one hand, high numbers of neoantigens could be identified in some patients, but only up to 20 neoantigens could be successfully synthesized for neoantigen vaccine preparation due to limited time and cost effectiveness. On the other hand, with the high heterogeneity in HCC, some tumor cells might not carry such neoantigens and thus could not be well recognized by activated T cells, resulting in growth superiority during neoantigen vaccination. Therefore, it is necessary to further strengthen the anti-recurrence efficacy and immune durability of neoantigen vaccines by optimizing the neoantigen vaccination strategy and/or combining with other treatment methods such as immune checkpoint inhibitors and tyrosine kinase inhibitors [24]. Recently, some reports have shown that neoantigen vaccine combined with PD-1 inhibitor in solid tumors such as melanoma and non-small cell carcinoma can further improve its anti-tumor efficacy [25], but its outcome in HCC remains to be further confirmed.

In summary, neoantigen vaccination is a safe, feasible and effective strategy for HCC anti-recurrence after radical surgery. Moreover, tracking personalized neoantigen mutations in ctDNA could provide comprehensive information for clinical response monitoring of neoantigen vaccination with high sensitivity and specificity, and thus help the clinical application for individualized medicine. However, this study still has the following shortcomings: firstly, due to time and funding constraints, personalized neoantigen tetramers were not custom-made for neoantigen-specific T cell analysis; secondly, due to limited sample size enrolled in this study, the corresponding findings will still need to be validated in large-scale clinical trials.

## Supplementary Information

**Additional file 1: Supplementary data.**

**Additional file 2: Supplementary Figure S1.** The profiling of DNA mutation and RNA editing identified in patient N18.

**Additional file 3: Supplementary Figure S2.** The Ex vivo IFN-γ ELISPOT responses for PBMCs stimulated by personalized neoantigen pools of patient N06 during neoantigen vaccinations.

**Additional file 4: Supplementary Figure S3.** The Ex vivo IFN-γ ELISPOT responses for PBMCs stimulated by personalized neoantigen pools of patient N13 during neoantigen vaccinations.

**Additional file 5: Supplementary Figure S4.** The Ex vivo IFN-γ ELISPOT responses for PBMCs stimulated by individual neoantigen peptide after neoantigen vaccinations (5 months) in 7 patients. The peptide in blue font, orange font and green font indicated as pool1, pool2, and pool3, respectively.

**Additional file 6: Supplementary Figure S5.** The mutation allele frequencies of other mutations in DNA level and RNA level between primary tumor and recurrent tumor.

**Additional file 7: Supplementary Figure S6.** The heatmap analysis of immune cell infiltration by transcriptomic data.

**Additional file 8: Supplementary Figure S7.** The profiling of other mutations detected in matched preoperative plasma.

**Additional file 9: Supplementary Figure S8.** The time-course demonstration of quantified levels of treated neoantigen mutations and other somatic mutations in patient N06(A) and N13(B), respectively. The small picture displays the dynamics of the average of mutation allele frequencies in treated neoantigen mutations and other somatic mutations during neoantigen vaccination and follow up.

**Additional file 10: Supplementary Table S1.** Baseline clinical characteristics between enrolled patients and PSM case-control patients.

**Additional file 11: Supplementary Table S2.** The detailed HLA type and neoantigen sequence for neoantigen vaccine preparation of each enrolled patients.

**Additional file 12: Supplementary Table S3.** Treatment-related adverse events in all enrolled patients.

**Additional file 13: Supplementary Table S4.** The dynamics of routine blood and biochemical tests in all enrolled patients during neoantigen vaccinations.

**Additional file 14: Supplementary Table S5.** The dynamic change in proportions of peripheral blood T lymphocyte subsets and serum levels of 6 cytokines in all enrolled patients during neoantigen vaccinations.

**Additional file 15: Supplementary Table S6.** New neoantigen mutations identified in recurrent tumor.

**Additional file 16: Supplementary Table S7.** The detailed ctDNA frequency of neoantigen mutations and other somatic mutations in enrolled patients during clinical course.

## Data Availability

The data used to support the findings of this study are included within the article. The raw sequencing data reported in this article has been deposited at Big Data center (Nucleic Acids Res 2018), under the accession number of HRA000096 that are publicly accessible at http://bigd.big.ac.cn/gsa.

## Abbreviations

HCC: Hepatocellular carcinoma
TACE: Transcatheter Arterial Chemoembolization
MHC: Major histocompatibility complex
MRD: Minimal residual disease
ctDNA: Circulating tumor DNA
RFS: Recurrence free survival
PBMCs: Peripheral blood mononuclear cells
TCR: T cell receptor
VAF: Variant allele frequency
