# Supplementary material for: Personalized neoantigen vaccine prevents postoperative recurrence in hepatocellular carcinoma patients with vascular invasion
Source: Mol Cancer. 2021 Dec 13;20:164. doi: 10.1186/s12943-021-01467-8 (PMC8667400; doi:10.1186/s12943-021-01467-8)
Supplement: Supplementary file 1 — Additional file 1: Supplementary data. [file 12943_2021_1467_MOESM1_ESM.docx]

**Supplementary data**

Personalized neoantigen vaccine prevents postoperative recurrence in hepatocellular carcinoma patients with vascular invasion

Zhixiong Cai ^#,1,2,3^, Xiaoping Su ^#,4^, Liman Qiu ^#,1,2,3^, Zhenli Li ^#,1,2,3^, Xiaolou Li ^1,2,3^, Xiuqing Dong ^1,2,3^, Fuqun Wei ^1,2,3^, Yang Zhou ^1,2,3^, Liuping Luo ^1,2,3^, Geng Chen ^1,2,3^, Hengkai Chen ^1,2,3^, Yingchao Wang ^1,2,3^, Yongyi Zeng ^*,1,2,3^ and Xiaolong Liu ^*,1,2,3^

^1^The United Innovation of Mengchao Hepatobiliary Technology Key Laboratory of Fujian Province, Mengchao Hepatobiliary Hospital of Fujian Medical University, Fuzhou 350025, P. R. China

^2^The Liver Center of Fujian Province, Fujian Medical University, Fuzhou 350025, P. R. China.

^3^Mengchao Med-X Center, Fuzhou University, Fuzhou 350116, P. R. China

^4^School of Basic Medicine, Wenzhou Medical University, Wenzhou Tea Mountain Higher Education Park, Wenzhou325027, P. R. China

***Corresponding authors**: Xiaolong Liu, Ph.D., E-mail: xiaoloong.liu@gmail.com. Yongyi Zeng, MD, Ph.D., E-mail: lamp197311@126.com.

**Supplementary method**

**Inclusion and exclusion criteria of enrolled patients**

The key inclusion criteria of enrolled patient is seen below: (1) aged 18 to 75 years old male and female, with serum bilirubin not higher than 1.5× upper limit of normal (ULN) and ALT or AST not higher than 2.0× ULN; (2) be diagnosed with resectable HCC or intrahepatic cholangiocarcinoma (CC) without any metastasis; (3) the existence of tumor thrombus in portal venous branch should be confirmed by histopathology or visible to the naked eye during the surgery. Key exclusion criteria included: (1) Patients with HIV infection, HCV infection, serious coronary artery disease or other diseases that the researchers consider not suitable to be included in this study; (2) Patients with history of bone marrow transplantation or organ transplantation; (3) Patients with any form of immunodeficiency or history of autoimmune disease; (4) Patients received prior treatment with any other immunotherapy within 1 month or have fewer than five identified actionable neoepitopes.

**IFN-γ Enzyme-Linked Immunospot (ELISpot) Assay**

To evaluate the immune response inducing by personalized neoantigen vaccines, ELISpot assays were performed for monitoring IFN-γ secretion of PBMCs simulated by neoantigen pools or each neoantigen individually at a series of time points, including pre-, post-vaccination, follow up time points, using Human IFN-γ ELISpotplus kit (MABTECH). Briefly, DC cells (2×10^4^ cells per well among enrolled patients) were firstly isolated from PBMCs and stimulated by neoantigen peptide pool or each synthesized neoantigen peptide for 24 hours. Then the remaining PBMCs (2×10^5^ cells per well among enrolled patients) were co-incubated with DC cells for another 24 hours in a 96-well plate. Then the corresponding spot detection was performed according to the standard protocol and the spots were imaged and counted by two experienced researchers under uniform standard. Meanwhile, autologous PBMCs stimulated by OKT3 antibody and by PBS-pulsed autologous DC cells were used as the positive control (PC) and the negative control (NC), respectively. The spot number of neoantigen peptide-stimulated PBMCs greater than twice in the negative control were considered with positive PBMC reactivity. Then the result was showed to the number of IFN-γ spots detected per 2×10^5^ PBMCs.

**Peripheral blood T lymphocyte subsets and** **cytokine assay**

Commercial TS cell count (Z6410004, Tongsheng Shidai Biotech, Beijing, China) was used to determine proportions of peripheral blood T lymphocyte subsets in patients' whole blood by flow cytometry according to their corresponding manufacturer's instructions. Commercial cytokine combination detection kit (Gan machine injection permit 20192400359, Saiji Biotechnology, Hangzhou, China) was used to determine serum levels of 6 cytokines (IL-2, IL-4, IL-6, IL-10, TNF-α and IFN-γ) by flow cytometry according to their corresponding manufacturer's instructions.

**Immunohistochemical staining**

Immunohistochemical staining of CD8 and granzyme B was performed using EliVision Plus staining technique. Briefly, after deparaffinized and rehydrated, a representative 5-μm section was firstly incubated in 1% hydrogen peroxide for 30 min to block endogenous peroxidase activity and then rehydrated. Afterwards, the slides were incubated in 0.1 mol/L EDTA (pH 9.0) for 3 min at high-pressure antigen retrieval. Subsequently, the slides were incubated with the primary antibody (CD8 mouse monoclonal antibody, 1:200 dilution, Proteintech; Granzyme B rabbit polyclonal antibody, 1:200 dilution, Abcolonal) at 4°C overnight and then incubated with secondary antibodies (Kit-9902; MXB, Fujian, China) at room temperature for another 2 h. Finally, the slides were subjected to DAB coloration and hematoxylin re-staining. The results were assessed by two experienced pathologists.

**Clonal evolution and immunophenoscore evaluation**

To assess the clonal evolution dynamics between primary tumor and recurrent tumor in patient N22, SciClone algorithm was conducted with the input of all the qualified somatic mutations (VAF >= 0.05) in copy number neutral region defined by cn.MOPS[^1^](#_ENREF_1)^,^ [^2^](#_ENREF_2). To show the immune landscape changes between primary tumor and recurrent tumor in patient N22 during neoantigen vaccination, the immunophenoscore diagrams were also derived with the RNA-seq data, using the source R code from Immunophenogram^[3](#_ENREF_3)^.

**Evaluation of immune cell infiltration**

Single sample Gene Set Enrichment analysis (ssGSEA) was performed to evaluate the relative level of immune cell infiltration, using the signature genes of 28 immune cell types obtained from Charoentong P’s study[^3^](#_ENREF_3) . The enrichment score of each immune cell type derived from ssGSEA analysis was used to represent the relative infiltration level of the 28 immune cell types.

**T cell receptor sequencing**

High-throughput TCR β-chain sequencing was performed by utilizing whole RNA extracted from tissue samples collected from patient N22, including primary tumor and recurrent tumor. A UID (unique identifier) was attached to each cDNA TCR molecule, allowing correction for sequencing errors and removing PCR duplicates. The filtered sequence reads were mapped to the TCR reference sequences provided by IMGT database[^4^](#_ENREF_4) using MiXCR software and amino acid sequences of CDR3 regions in the TCRβ-chain were also defined[^5^](#_ENREF_5). New TCR clones was defined as the clones which were absent or with a frequency of less than 0.005% of all clones sequenced in the primary tumor sample, and were with a frequency of no less than 0.5% of all clones sequenced in the recurrent tumor after vaccination.

**ctDNA sequencing**

Cell-free DNA (cfDNA) was extracted from serial plasma samples of enrolled patients using QIAamp Circulating Nucleic Acid Kit (QIAGEN) and then subjected to library preparation using NanoPrep DNA library Preparation Kit with duplex UMI Adapters. Subsequently, the cfDNA library was further enriched by using in-house customized panel containing personalized somatic mutations of enrolled patients and then sequenced by Fulgent. Co., Ltd. on Illumina Novaseq 6000 platform with raw coverage depths of 10000× (paired end, 150bp). The UMI sequencing data were processed using ConsensusCruncher to amalgamate reads generated from the same DNA template into a consensus sequence[^6^](#_ENREF_6). The final output of single-strand consensus sequences + singleton correction bam files were used in downstream analysis as recommended. To accurately detect mutation with relatively low variant frequency, we deployed bam2R to extract the mutation frequency and read all personalized somatic mutations identified in tumor tissues. Somatic mutations in ctDNA were further filtered under following criteria: (1) minimal of 3 mutant reads were observed in preoperative plasma; (2) mutation frequency was greater than or equal to 0.5% in preoperative plasma; (3) overall sequencing reads were greater than or equal to 500 in all following plasma.

**Reference**

1. Klambauer G, Schwarzbauer K, Mayr A et al. cn.MOPS: mixture of Poissons for discovering copy number variations in next-generation sequencing data with a low false discovery rate. *Nucleic Acids Res*. 2012; 40: e69.

2. Miller CA, White BS, Dees ND et al. SciClone: inferring clonal architecture and tracking the spatial and temporal patterns of tumor evolution. *PLoS Comput Biol*. 2014; 10: e1003665.

3. Charoentong P, Finotello F, Angelova M et al. Pan-cancer Immunogenomic Analyses Reveal Genotype-Immunophenotype Relationships and Predictors of Response to Checkpoint Blockade. *Cell Rep*. 2017; 18: 248-262.

4. Lefranc M-P, Giudicelli V, Duroux P et al. IMGT®, the international ImMunoGeneTics information system® 25 years on. *Nucleic acids research*. 2015; 43: D413-D422.

5. Bolotin DA, Poslavsky S, Mitrophanov I et al. MiXCR: software for comprehensive adaptive immunity profiling. *Nature methods*. 2015; 12: 380-381.

6. Wang TT, Abelson S, Zou J et al. High efficiency error suppression for accurate detection of low-frequency variants. *Nucleic acids research*. 2019; 47: e87-e87.
