# Supplementary figures and images for: Personalized neoantigen vaccine prevents postoperative recurrence in hepatocellular carcinoma patients with vascular invasion
Source: Mol Cancer. 2021 Dec 13;20:164. doi: 10.1186/s12943-021-01467-8 (PMC8667400; doi:10.1186/s12943-021-01467-8)

# Supplementary Figure S1

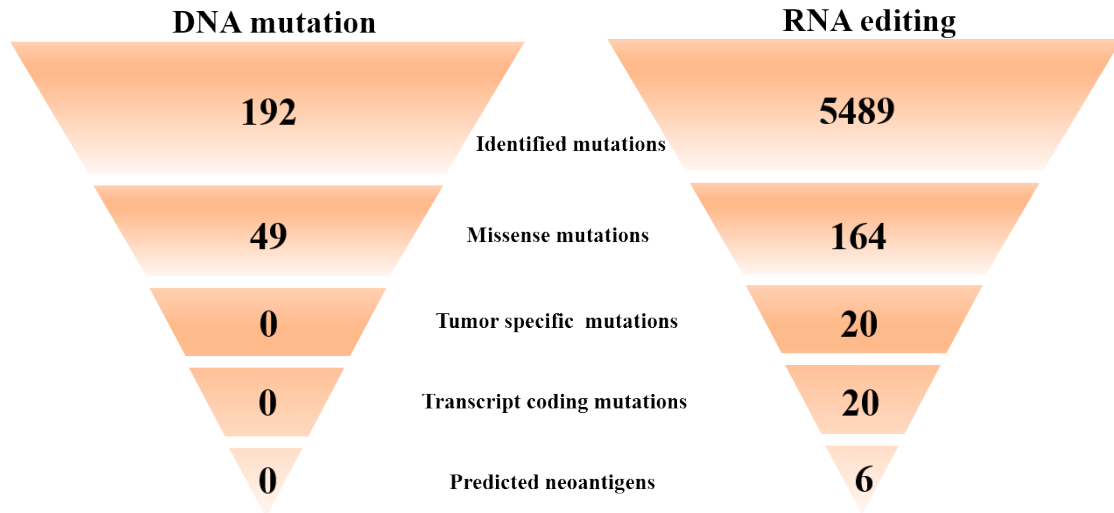

Supplement: Supplementary file 2 — Additional file 2: Supplementary Figure S1. The profiling of DNA mutation and RNA editing identified in patient N18. [file 12943_2021_1467_MOESM2_ESM.pdf]

# Supplementary Figure S2

N06

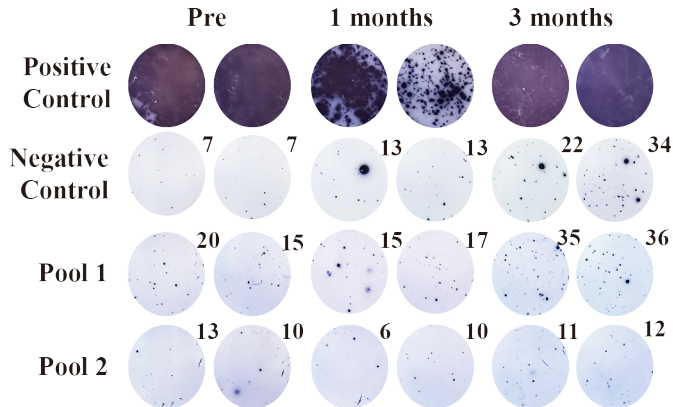

Supplement: Supplementary file 3 — Additional file 3: Supplementary Figure S2. The Ex vivo IFN-γ ELISPOT responses for PBMCs stimulated by personalized neoantigen pools of patient N06 during neoantigen vaccinations. [file 12943_2021_1467_MOESM3_ESM.pdf]

## Supplementary Figure S3

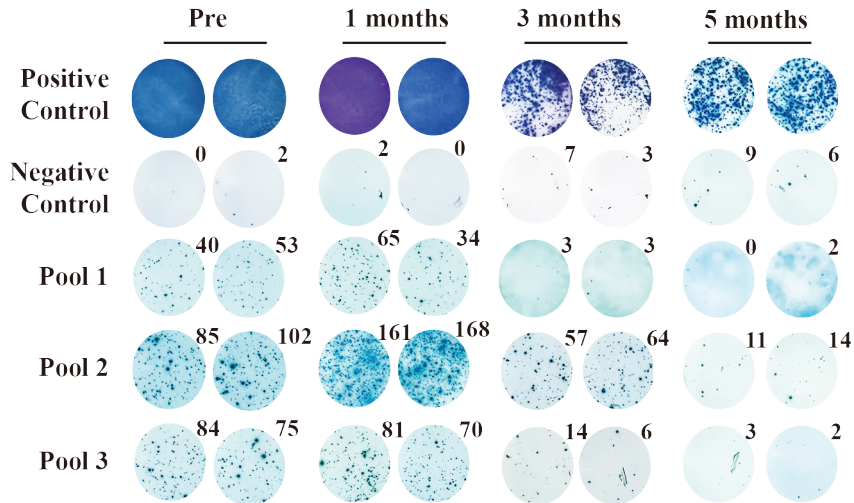

Supplement: Supplementary file 4 — Additional file 4: Supplementary Figure S3. The Ex vivo IFN-γ ELISPOT responses for PBMCs stimulated by personalized neoantigen pools of patient N13 during neoantigen vaccinations. [file 12943_2021_1467_MOESM4_ESM.pdf]

# Supplementary Figure S4

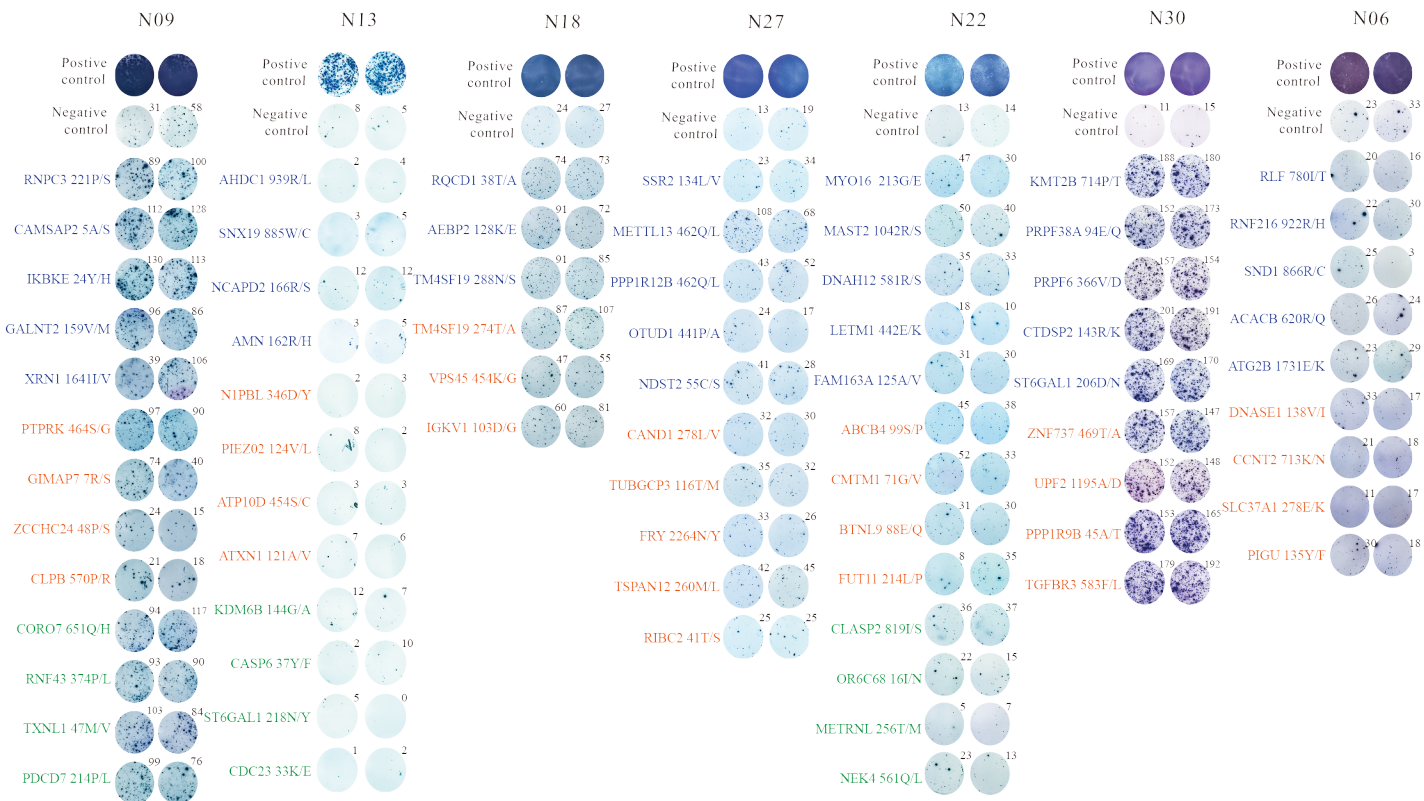

Supplement: Supplementary file 5 — Additional file 5: Supplementary Figure S4. The Ex vivo IFN-γ ELISPOT responses for PBMCs stimulated by individual neoantigen peptide after neoantigen vaccinations (5 months) in 7 patients. The peptide in blue font, orange font and green font indicated as pool1, pool2, and pool3, respectively. [file 12943_2021_1467_MOESM5_ESM.pdf]

## Supplementary Figure S5

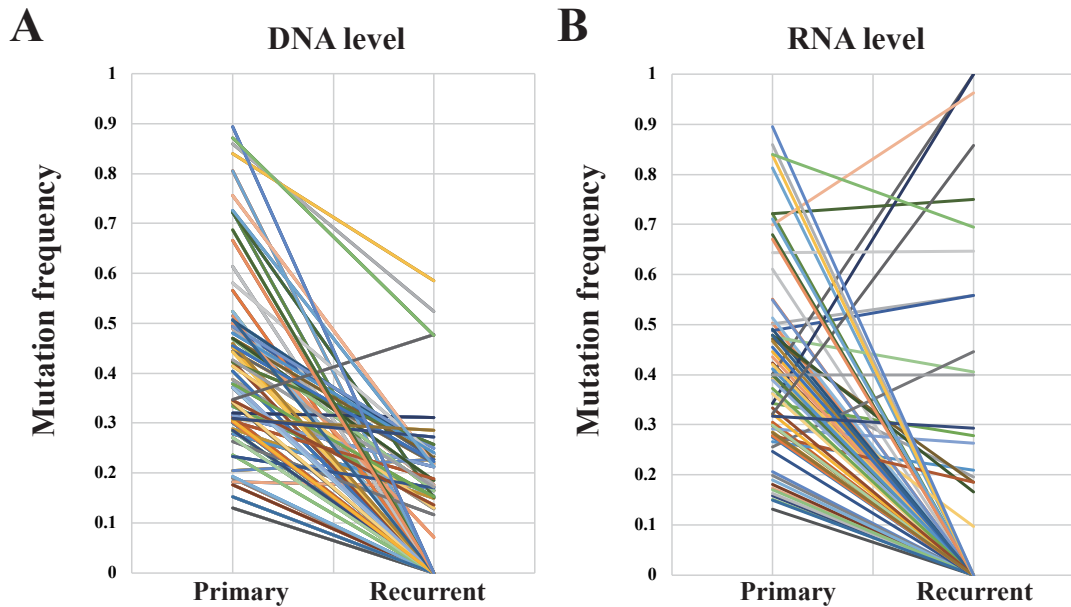

Supplement: Supplementary file 6 — Additional file 6: Supplementary Figure S5. The mutation allele frequencies of other mutations in DNA level and RNA level between primary tumor and recurrent tumor. [file 12943_2021_1467_MOESM6_ESM.pdf]

# Supplementary Figure S6

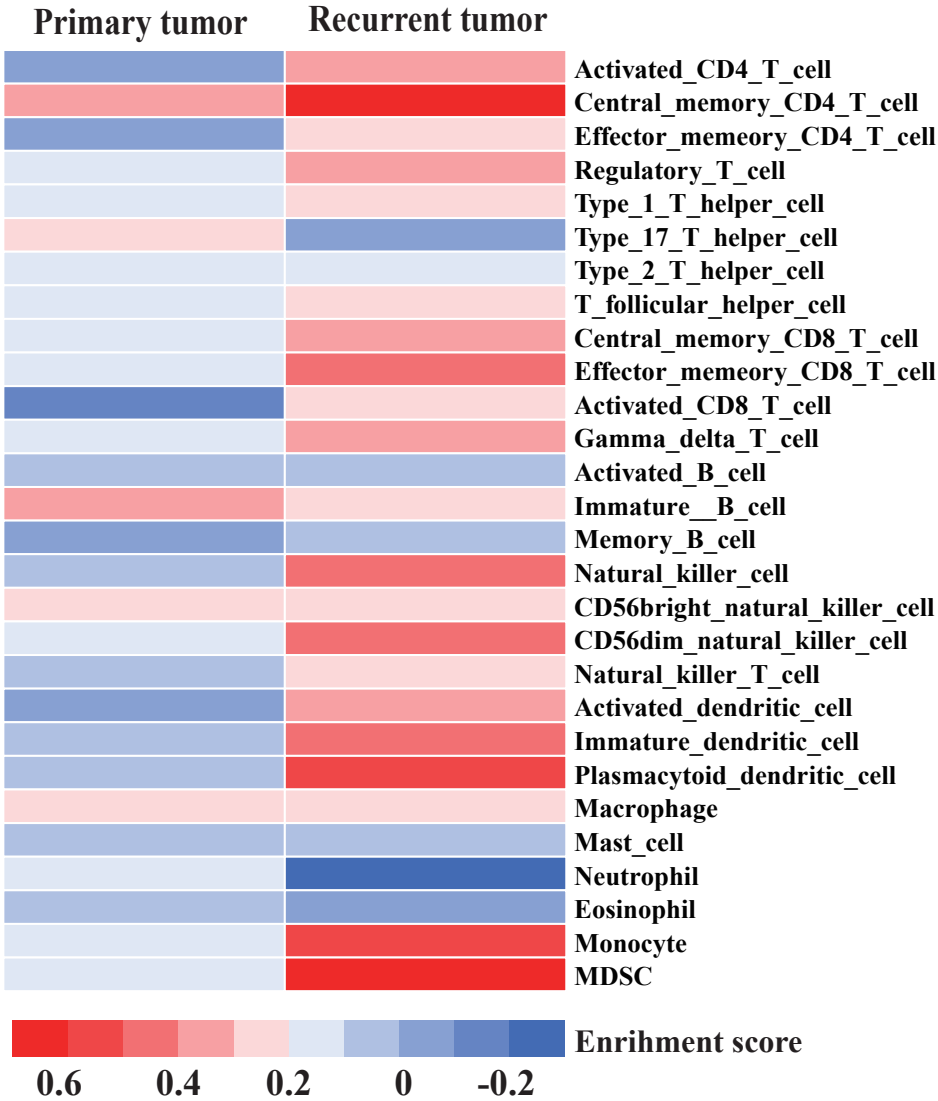

Supplement: Supplementary file 7 — Additional file 7: Supplementary Figure S6. The heatmap analysis of immune cell infiltration by transcriptomic data. [file 12943_2021_1467_MOESM7_ESM.pdf]

Supplementary Figure S8

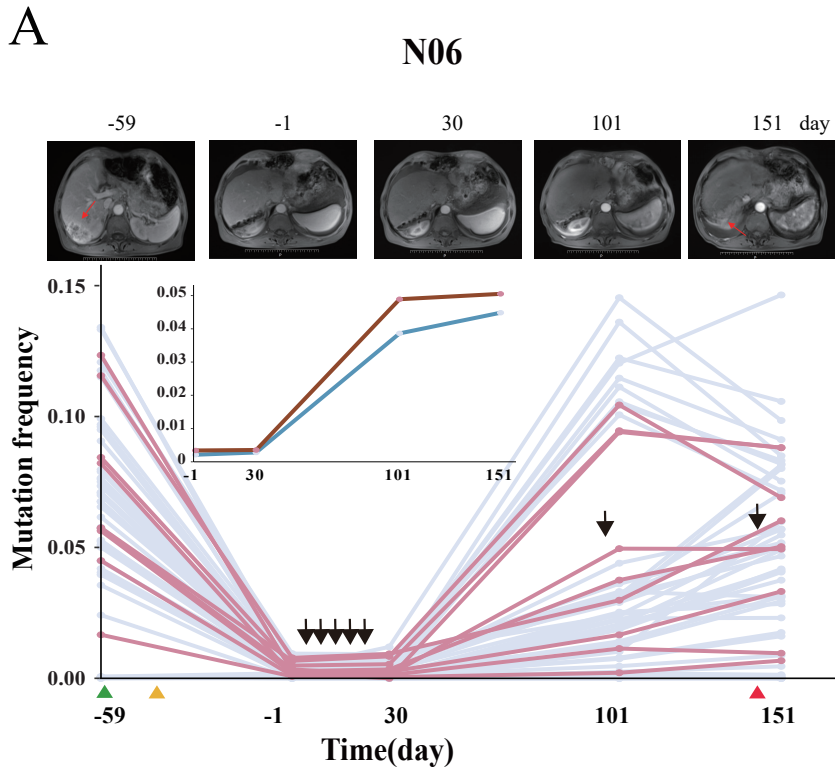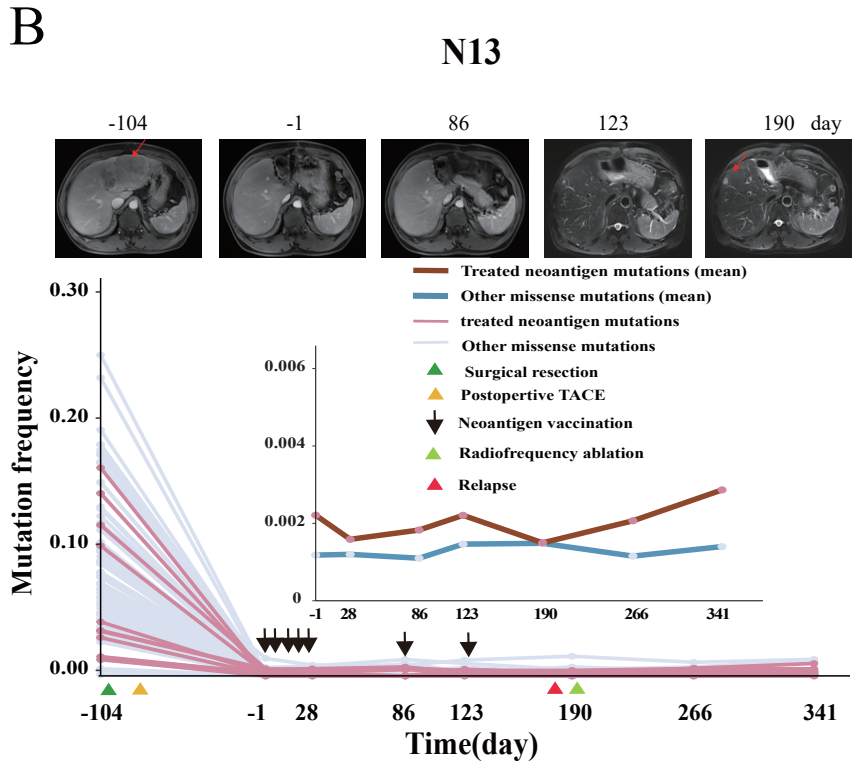

Supplement: Supplementary file 9 — Additional file 9: Supplementary Figure S8. The time-course demonstration of quantified levels of treated neoantigen mutations and other somatic mutations in patient N06(A) and N13(B), respectively. The small picture displays the dynamics of the average of mutation allele frequencies in treated neoantigen mutations and other somatic mutations during neoantigen vaccination and follow up. [file 12943_2021_1467_MOESM9_ESM.pdf]
