# Supplementary material for: Personalized neoantigen vaccine prevents postoperative recurrence in hepatocellular carcinoma patients with vascular invasion
Source: Mol Cancer. 2021 Dec 13;20:164. doi: 10.1186/s12943-021-01467-8 (PMC8667400; doi:10.1186/s12943-021-01467-8)

Supplementary Figure S7

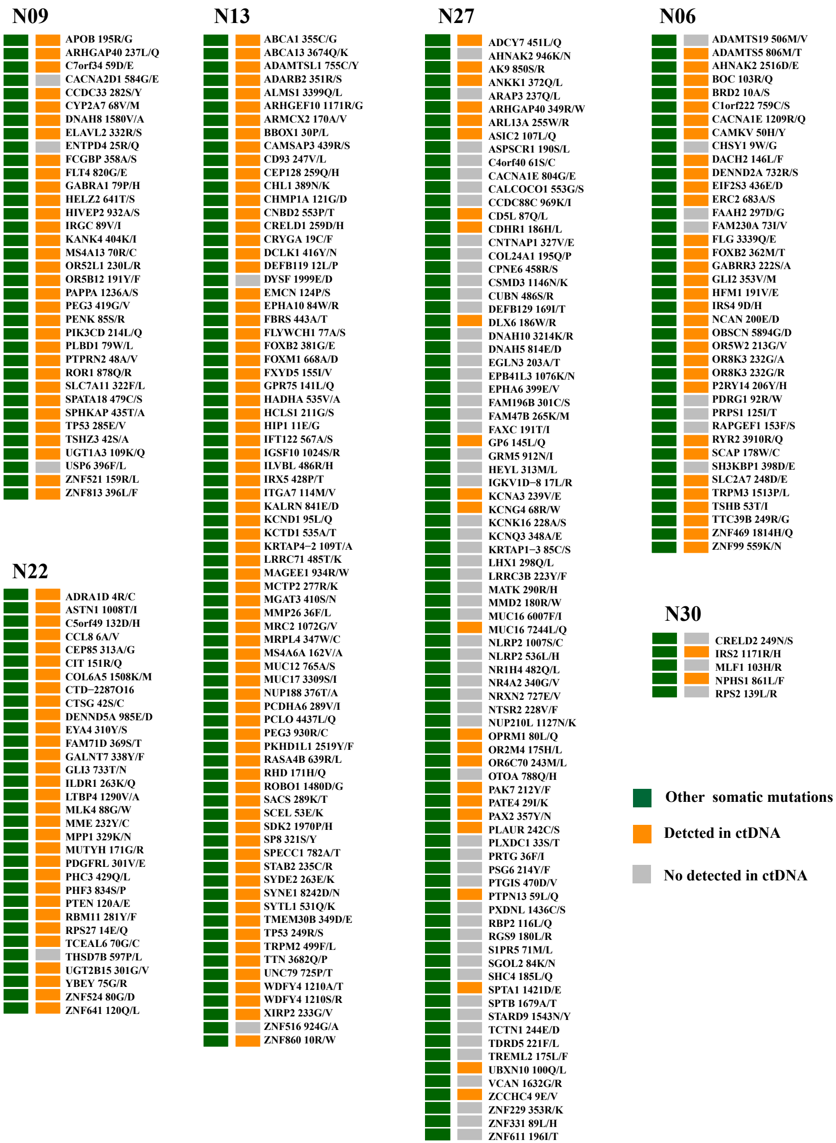

Supplement: Supplementary file 8 — Additional file 8: Supplementary Figure S7. The profiling of other mutations detected in matched preoperative plasma. [file 12943_2021_1467_MOESM8_ESM.pdf]
