# Supplementary material for: Personalized neoantigen vaccine prevents postoperative recurrence in hepatocellular carcinoma patients with vascular invasion
Source: Mol Cancer. 2021 Dec 13;20:164. doi: 10.1186/s12943-021-01467-8 (PMC8667400; doi:10.1186/s12943-021-01467-8)
Supplement: Supplementary file 10 — Additional file 10: Supplementary Table S1. Baseline clinical characteristics between enrolled patients and PSM case-control patients. [file 12943_2021_1467_MOESM10_ESM.docx]

**Supplementary Table S1. Baseline clinical characteristics of enrolled patients and PSM control patients.**

| **Clinical characteristics** | **Patients without neoantigen vaccination**  **(PSM, n=20)** | **Patients with neoantigen vaccination**  **(n=10)** | ***p*-value** ^a^ |
| --- | --- | --- | --- |
| **Tumor pathological type** | | |  |
| HCC | 17 | 5 | 0.078 |
| HCC-ICC | 3 | 5 |  |
| **Tumor differentiation** | | |  |
| Poor | 12 | 6 | 1.000 |
| Moderate | 8 | 4 |  |
| **Child-Pugh grading** | |  |  |
| A stage | 18 | 9 | 1.000 |
| B stage | 2 | 1 |  |
| **Tumor number** | | |  |
| Single | 15 | 7 | 1.000 |
| Multiple | 5 | 3 |  |
| **Maximal tumor size, cm** |  |  |  |
| <5 | 2 | 2 | 0.584 |
| ≥5 | 18 | 8 |  |
| **TNM stage** | | |  |
| II | 0 | 1 | 0.333 |
| III | 20 | 9 |  |

a. Fisher’s exact test
