# Supplementary material for: Personalized neoantigen vaccine prevents postoperative recurrence in hepatocellular carcinoma patients with vascular invasion
Source: Mol Cancer. 2021 Dec 13;20:164. doi: 10.1186/s12943-021-01467-8 (PMC8667400; doi:10.1186/s12943-021-01467-8)
Supplement: Supplementary file 12 — Additional file 12: Supplementary Table S3. Treatment-related adverse events in all enrolled patients. [file 12943_2021_1467_MOESM12_ESM.docx]

**Supplementary Table S3. Treatment-related adverse events in all enrolled patients.**

| Event | All enrolled patients (n=10) | | Adverse reactions subsided without clinical intervention |
| --- | --- | --- | --- |
|  | **Grade 1-2** | **Grade 3-4** |  |
| Injection site reaction |  |  |  |
| Local pain | 5 (50%) | 0 | 5 (100%) |
| Local pruritus | 0 | 0 | 0 |
| Redness and swelling | 0 | 0 | 0 |
| Skin |  |  |  |
| Maculopapule | 0 | 0 | 0 |
| Rash | 0 | 0 | 0 |
| Digestive system |  |  |  |
| Dry mouth | 0 | 0 | 0 |
| Nausea | 0 | 0 | 0 |
| Vomiting | 0 | 0 | 0 |
| Diarrhea | 0 | 0 | 0 |
| Constipation | 0 | 0 | 0 |
| Respiratory system |  |  |  |
| Cough | 0 | 0 | 0 |
| Dyspnea | 0 | 0 | 0 |
| Other |  |  |  |
| Fever | 0 | 0 | 0 |
| Chills | 0 | 0 | 0 |
| Fatigue | 5 (50%) | 0 | 5 (100%) |
| Headache | 0 | 0 | 0 |
| Dizziness | 0 | 0 | 0 |
| Anemia | 0 | 0 | 0 |
