# Supplementary material for: Personalized neoantigen vaccine prevents postoperative recurrence in hepatocellular carcinoma patients with vascular invasion
Source: Mol Cancer. 2021 Dec 13;20:164. doi: 10.1186/s12943-021-01467-8 (PMC8667400; doi:10.1186/s12943-021-01467-8)
Supplement: Supplementary file 13 — Additional file 13: Supplementary Table S4. The dynamics of routine blood and biochemical tests in all enrolled patients during neoantigen vaccinations. [file 12943_2021_1467_MOESM13_ESM.docx]

**Supplementary table S4. The dynamics of routine blood and biochemical tests in all enrolled patients during neoantigen vaccinations**

| **Time** | **N06** | | | | **N09** | | | | | **N13** | | | | | **N18** | | | | | **N22** | | | | |
| --- | --- | --- | --- | --- | --- | --- | --- | --- | --- | --- | --- | --- | --- | --- | --- | --- | --- | --- | --- | --- | --- | --- | --- | --- |
| **Items** | Pre- | 30d | 90d | 140d | Pre- | 30d | 90d | 140d | V1 | Pre- | 30d | 90d | 140d | V1 | Pre- | 30d | 90d | 140d | V1 | Pre- | 30d | 90d | 140d | V1 |
| **Child-Puge** | A | A | A | A | A | A | A | A | A | A | A | A | A | A | A | A | A | A | A | A | A | A | A | A |
| **ALT (U/L)** | 40.0 | 50.0 | 41.0 | 51.0 | 46.0 | 49.0 | 21.0 | 21.0 | 21.0 | 40.0 | 30.0 | 38.0 | 24.0 | 39.0 | 36.0 | 42.0 | 44.0 | 50.0 | 42.0 | 30.0 | 31.0 | 30.0 | 26.0 | 25.0 |
| **AST (U/L)** | 47.0 | 55.0 | 43.0 | 47.0 | 92.0 | 55.0 | 48.0 | 42.0 | 42.0 | 25.0 | 21.0 | 21.0 | 19.0 | 24.0 | 34.0 | 33.0 | 32.0 | 36.0 | 40.0 | 27.0 | 29.0 | 26.0 | 22.0 | 23.0 |
| **sCr (μmol/L)** | 77.0 | 71.0 | 66.0 | 63.0 | 90.0 | 65.0 | 65.0 | 63.0 | 65.0 | 94.0 | 70.0 | 75.0 | 67.0 | 68.0 | 63.0 | 73.0 | 75.0 | 71.0 | 74.0 | 72.0 | 69.0 | 76.0 | 77.0 | 72.0 |
| **CK (U/L)** | 45.0 | 51.0 | 70.0 | 60.0 | 159.0 | 212.0 | 124.0 | 158.0 | 230.0 | 49.0 | 78.0 | 61.0 | 70.0 | 77.0 | 98.0 | 72.0 | 98.0 | 104.0 | 129.0 | 90.0 | 115.0 | 106.0 | 98.0 | 94.0 |
| **CK-MB (U/L)** | 26.0 | 26.6 | 21.8 | 24.9 | 62.4 | 20.2 | 21.6 | 32.6 | 28.0 | 10.1 | 7.7 | 13.2 | 11.1 | 17.1 | 21.0 | 22.2 | 23.1 | 16.7 | 21.9 | 14.3 | 22.4 | 19.1 | 13.4 | 11.2 |
| **LDH (U/L)** | 259 | 228 | 228 | 203 | 432 | 233 | 194 | 211 | 213 | 137 | 139 | 163 | 153 | 180 | 147 | 139 | 187 | 139 | 180 | 166 | 216 | 209 | 149 | 179 |
| **CRP (mg/L)** | - | - | - | - | - | - | - | - | - | 3.97 | 6.90 | 3.26 | 7.75 | 2.08 | 1.52 | 1.01 | 2.27 | ＜0.75 | ＜0.75 | ＜0.75 | 0.61 | ＜0.75 | ＜0.75 | 5.95 |
| **WBC (*10^9/L)** | 3.91 | 3.84 | 5.07 | 4.44 | 2.25 | 2.79 | 2.27 | 2.83 | 2.44 | 4.68 | 5.17 | 5.29 | 5.05 | 4.43 | 4.443 | 4.02 | 4.8 | 4.32 | 4.66 | 4.79 | 5.7 | 5.69 | 6.08 | 6.2 |
| **Hb (g/L)** | 118 | 118 | 112 | 116 | 173 | 136 | 138 | 140 | 139 | 136 | 148 | 151 | 125 | 150 | 139 | 143 | 152 | 156 | 160 | 144 | 139 | 141 | 138 | 143 |
| **PLT(*10^9/L)** | 119 | 122 | 125 | 142 | 85 | 132 | 82 | 85 | 67 | 182 | 196 | 172 | 184 | 177 | 76 | 79 | 94 | 83 | 92 | 165 | 152 | 162 | 186 | 169 |
| **PT (s)** | 13.8 | 14.8 | 15.0 | 14.3 | 14.0 | 14.3 | 14.9 | 14.5 | 13.3 | 13.1 | 13.4 | 12.5 | 12.8 | 12.4 | 13.5 | 12.9 | 12.0 | 12.8 | 13.0 | 12.4 | 12.9 | 13.0 | 12.2 | 12.6 |
| **APTT (s)** | 34.7 | 36.9 | 34.8 | 30.4 | 38.6 | 38.6 | 39.0 | 37.8 | 42.5 | 38.7 | 36.9 | 35.6 | 38.9 | 35.8 | 35.2 | 36.5 | 37.7 | 33.4 | 33.3 | 42.8 | 36.6 | 37.2 | 39.6 | 36.6 |
| **TT (s)** | 16.3 | 17.5 | 16.7 | 16.5 | 17.7 | 18.1 | 17.1 | 17.0 | 18.5 | 16.1 | 16.1 | 16.2 | 15.9 | 16.9 | 17.3 | 17.7 | 16.4 | 17.1 | 16.9 | 15.7 | 17.3 | 17.8 | 16.1 | 18.2 |
| **FIB (g/L)** | 2.65 | 2.11 | 2.21 | 2.84 | 3.32 | 2.54 | 2.34 | 2.14 | 2.18 | 2.93 | 3.92 | 2.93 | 3.83 | 2.69 | 2.21 | 2.34 | 2.46 | 2.46 | 2.68 | 2.93 | 2.57 | 2.23 | 3.90 | 3.90 |
| **Urine-rt** | (-) | (-) | (-) | (-) | (-) | (-) | (-) | (-) | (-) | (-) | (-) | (-) | (-) | (-) | (-) | (-) | a | (-) | (-) | (-) | (-) | (-) | (-) | (-) |
| **stool-rt** | b | b | (-) | (-) | (-) | (-) | (-) | (-) | (-) | (-) | (-) | (-) | (-) | (-) | (-) | (-) | (-) | (-) | (-) | (-) | (-) | (-) | (-) | (-) |
| **ECOG** | 0 | 0 | 0 | 0 | 0 | 0 | 0 | 0 | 0 | 0 | 0 | 0 | 0 | 0 | 0 | 0 | 0 | 0 | 0 | 0 | 0 | 0 | 0 | 0 |

**Supplementary table S4. The dynamics of routine blood and biochemical tests in all enrolled patients during neoantigen vaccinations(continue)**

| **Time** | **N24** | | | | **N25** | | | | **N27** | | | | | **N30** | | | | | **N31** | | | |
| --- | --- | --- | --- | --- | --- | --- | --- | --- | --- | --- | --- | --- | --- | --- | --- | --- | --- | --- | --- | --- | --- | --- |
| **Items** | Pre- | 30d | 90d | 140d | Pre- | 30d | 90d | 140d | Pre- | 30d | 90d | 140d | V1 | Pre- | 30d | 90d | 140d | V1 | Pre- | 30d | 90d | 140d |
| **Child-Puge** | A | A | - | - | A | A | - | - | A | A | A | A | A | A | A | A | A | A | A | A | - | - |
| **ALT (U/L)** | 25.0 | 21.0 | - | - | 18.0 | 17.0 | - | - | 28.0 | 34.0 | 36.0 | 26.0 | 31.0 | 23.0 | 25.0 | 31 | 28.0 | 25.0 | 44.0 | 36.0 | - | - |
| **AST (U/L)** | 23.0 | 22.0 | - | - | 21.0 | 21.0 | - | - | 25.0 | 21.0 | 21.0 | 19.0 | 24.0 | 34.0 | 33.0 | 32.0 | 36.0 | 49.0 | 27.0 | 29.0 | - | - |
| **sCr (μmol/L)** | 62.0 | 73.0 | - | - | 75.0 | 72.0 | - | - | 84.0 | 84.0 | 87.0 | 74.0 | 72.0 | 60.0 | 61.0 | 70.0 | 67.0 | 64.0 | 68.0 | 82.0 | - | - |
| **CK (U/L)** | 91.0 | 175.0 | - | - | 51.0 | 48.0 | - | - | 101.0 | 125.0 | 78.0 | 87.0 | 90.0 | 131.0 | 201.0 | 150.0 | 137.0 | 154.0 | 81.0 | 76.0 | - | - |
| **CK-MB (U/L)** | 18.9 | 20.0 | - | - | 14.2 | 11.8 | - | - | 12.4 | 16.1 | 19.6 | 17.7 | 37.5 | 37.2 | 47.3 | 38.7 | 45.3 | 38.9 | 9.3 | 20.0 | - | - |
| **LDH (U/L)** | 136 | 169 | - | - | 261 | 178 | - | - | 176 | 149 | 145 | 168 | 193 | 157 | 164 | 192 | 175 | 185 | 165 | 189 | - | - |
| **CRP (mg/L)** | 1.65 | 2.85 | - | - | 0.74 | 1.95 | - | - | ＜0.75 | ＜0.75 | ＜0.75 | ＜0.75 | ＜0.75 | ＜0.75 | 1.00 | 0.89 | ＜0.75 | ＜0.75 | ＜0.75 | 1.58 | - | - |
| **WBC (*10^9/L)** | 4.58 | 4.65 | - | - | 4.54 | 4.34 | - | - | 2.95 | 3.69 | 2.72 | 2.93 | 3.50 | 3.09 | 4.05 | 3.78 | 4.56 | 4.37 | 5.68 | 6.81 | - | - |
| **Hb (g/L)** | 150 | 153 | - | - | 130 | 120 | - | - | 152 | 157 | 151 | 146 | 150 | 138 | 143 | 145 | 151 | 147 | 154 | 169 | - | - |
| **PLT(*10^9/L)** | 83 | 93 | - | - | 206 | 154 | - | - | 128 | 130 | 99 | 94 | 110 | 83 | 107 | 100 | 97 | 121 | 160 | 177 | - | - |
| **PT (s)** | 14.4 | 13.4 | - | - | 12.7 | 12.2 | - | - | 12.8 | 12.7 | 13.2 | 13.5 | 12.5 | 12.7 | 13.0 | 13.7 | 12.5 | 12.9 | 13.4 | 13.2 | - | - |
| **APTT (s)** | 35.2 | 34.2 | - | - | 29.0 | 39.0 | - | - | 38.4 | 37.6 | 36.6 | 32.9 | 37.1 | 33.2 | 35.9 | 34.9 | 32.9 | 33.0 | 36.4 | 37.9 | - | - |
| **TT (s)** | 16.5 | 16.2 | - | - | 15.5 | 16.1 | - | - | 16.3 | 16.9 | 16.0 | 16.3 | 16.2 | 16.7 | 14.9 | 17.3 | 16.8 | 16.9 | 16.4 | 17.2 | - | - |
| **FIB (g/L)** | 1.80 | 2.49 | - | - | 2.92 | 3.02 | - | - | 2.30 | 2.66 | 2.70 | 2.54 | 2.55 | 3.27 | 3.85 | 3.77 | 3.21 | 3.00 | 3.44 | 3.84 | - | - |
| **Urine-rt** | (-) | (-) | - | - | (-) | (-) | - | - | (-) | (-) | (-) | (-) | (-) | (-) | (-) | (-) | (-) | (-) | (-) | (-) | - | - |
| **stool-rt** | (-) | (-) | - | - | (-) | (-) | - | - | (-) | (-) | (-) | (-) | (-) | (-) | (-) | (-) | (-) | (-) | (-) | (-) | - | - |
| **ECOG** | 0 | 0 | - | - | 0 | 0 | - | - | 0 | 0 | 0 | 0 | 0 | 0 | 0 | 0 | 0 | 0 | 0 | 0 | - | - |

- No detection，

（-） Negative，

a Urine-rt: Urine protein is weakly positive.

b Stool-rt: Fecal occult blood is weakly positive.

Acronym: ALT (alanine aminotransferase), AST (aspartate aminotransferase), sCr (Serum creatinine), CK (Creatine Kinase), CK-MB (Creatine kinase isoenzymes), LDH (Lactate dehydrogenase), CRP (C-reactive Protein), WBC (White Blood Cell), Hb (Hemoglobin), PLT (Platelet), PT (Prothrombin time), APTT (Activated partial thromboplastin time), TT (Thrombin time) , FIB (Fibrinogen).
