# Supplementary material for: Personalized neoantigen vaccine prevents postoperative recurrence in hepatocellular carcinoma patients with vascular invasion
Source: Mol Cancer. 2021 Dec 13;20:164. doi: 10.1186/s12943-021-01467-8 (PMC8667400; doi:10.1186/s12943-021-01467-8)
Supplement: Supplementary file 14 — Additional file 14: Supplementary Table S5. The dynamic change in proportions of peripheral blood T lymphocyte subsets and serum levels of 6 cytokines in all enrolled patients during neoantigen vaccinations. [file 12943_2021_1467_MOESM14_ESM.docx]

**Supplementary Table S5. The dynamic change in proportions of peripheral blood T lymphocyte subsets and serum levels of 6 cytokines in all enrolled patients during neoantigen vaccinations**

| **Items**  **Time** | | **CD3**  **(%)** | **CD4**  **(%)** | **CD8**  **(%)** | **IL-2**  **(pg/ml)** | **IL-4**  **(pg/ml)** | **IL-6**  **(pg/ml)** | **IL-10**  **(pg/ml)** | **TNF-α**  **(pg/ml)** | **IFN-γ**  **(pg/ml)** |
| --- | --- | --- | --- | --- | --- | --- | --- | --- | --- | --- |
| **N06** | Pre- | 65.55 | 40.12 | 11.37 | 0.69 | 1.38 | 4.97 | 1.61 | 0.62 | 0.18 |
|  | 30d | 68.44 | 44.15 | 14.75 | 0.81 | 1.51 | 4.92 | 1.57 | 0.45 | 0.11 |
|  | 90d | 66.67 | 39.30 | 12.51 | 0.67 | 1.64 | 8.45 | 0.91 | 0.49 | 0.02 |
|  | 140d | 64.91 | 38.84 | 15.35 | 0.67 | 0.56 | 9.79 | 1.21 | 0.28 | 0.7 |
| **N09** | Pre- | 49.68 | 31.99 | 16.53 | 0.67 | 0.68 | 17.58 | 1.91 | 0.96 | 0.66 |
|  | 30d | 61.90 | 42.02 | 20.97 | 0.69 | 1.01 | 4.28 | 2.21 | 0.05 | 0.57 |
|  | 90d | 56.56 | 39.11 | 16.25 | 0.67 | 1.34 | 3.69 | 2.11 | 0.57 | 0.47 |
|  | 140d | 57.64 | 41.58 | 16.80 | 0.32 | 1.01 | 9.65 | 2.62 | 0.08 | 0.73 |
|  | V1 | 62.53 | 43.55 | 17.18 | 0.63 | 1.13 | 4.1 | 2.11 | 0.36 | 0.7 |
| **N13** | Pre- | 63.51 | 32.63 | 28.55 | 8.08 | 2.84 | 4.38 | 2.83 | 34.59 | 0.37 |
|  | 30d | 68.24 | 39.10 | 27.66 | 3.7 | 4.08 | 5.97 | 1.41 | 6.89 | 1.37 |
|  | 90d | 69.79 | 39.17 | 28.64 | 5.35 | 3.99 | 4.97 | 1.61 | 12.16 | 0.43 |
|  | 140d | 67.42 | 41.55 | 27.50 | 3.2 | 3.25 | 11.08 | 1.51 | 7.27 | 0.47 |
|  | V1 | 62.76 | 42.20 | 25.10 | 3.83 | 2.84 | 1.97 | 1.47 | 8.55 | 0.34 |
| **N18** | Pre- | 80.74 | 36.99 | 28.86 | 0.63 | 1.22 | 5.01 | 1.61 | 1.33 | 0.5 |
|  | 30d | 81.41 | 40.22 | 24.89 | 0.5 | 1.17 | 4.15 | 1.41 | 0.62 | 0.24 |
|  | 90d | 77.36 | 35.18 | 23.79 | 0.11 | 0.96 | 4.47 | 2.08 | 0.28 | 0.6 |
|  | 140d | 77.56 | 37.28 | 22.86 | 0.5 | 1.22 | 4.19 | 1.44 | 0 | 0.43 |
|  | V1 | 65.62 | 19.68 | 13.72 | 0.76 | 1.26 | 4.1 | 1.08 | 0.24 | 0.37 |
| **N22** | Pre- | 65.65 | 40.84 | 22.94 | 0.52 | 1.77 | 3.06 | 1.24 | 0.24 | 0.43 |
|  | 30d | 63.59 | 37.29 | 24.45 | 0.67 | 1.26 | 4.92 | 1.04 | 1.01 | 0.37 |
|  | 90d | 66.85 | 40.88 | 24.76 | 0.54 | 1.86 | 2.11 | 1.04 | 0.32 | 0.4 |
|  | 140d | 64.92 | 38.81 | 22.29 | 0.83 | 1.64 | 3.19 | 1.34 | 0.7 | 0.4 |
| **N24** | Pre- | 77.32 | 35.59 | 38.14 | 1.54 | 4.79 | 5.15 | 2.21 | 2.37 | 0.9 |
|  | 30d | 75.38 | 32.83 | 37.66 | 1.97 | 5.99 | 15.55 | 2.55 | 4.31 | 0.21 |
| **N25** | Pre- | 60.19 | 37.82 | 18.33 | 1.59 | 0.88 | 11.58 | 2.31 | 3.22 | 1.23 |
|  | 30d | 61.46 | 34.94 | 21.81 | 1.47 | 2.17 | 19.75 | 2.49 | 2.23 | 0.66 |
| **N27** | Pre- | 61.60 | 25.58 | 35.02 | 12.49 | 17.04 | 17.49 | 18.45 | 31.04 | 9.41 |
|  | 30d | - | - | - | 10.71 | 12.01 | 16.47 | 16.47 | 25.26 | 9.8 |
|  | 90d | 55.89 | 27.81 | 27.36 | 9.19 | 13.49 | 11.58 | 14.58 | 19.92 | 4.83 |
|  | 140d | 52.08 | 29.14 | 25.28 | 10.47 | 13.8 | 10.71 | 12.8 | 23.85 | 11.72 |
| **N30** | Pre- | 61.60 | 25.58 | 18.75 | 0.76 | 1.69 | 3.47 | 1.44 | 0.45 | 0.31 |
|  | 30d | - | - | - | 1.01 | 0.6 | 5.15 | 0.82 | 0.01 | 0.66 |
|  | 90d | 55.89 | 27.81 | 19.35 | - | - | - | - | - | - |
|  | 140d | 52.08 | 29.14 | 18.75 | - | - | - | - | - | - |
| **N31** | Pre- | 52.20 | 26.44 | 20.23 | 0.16 | 1.47 | 2.47 | 0.75 | 0.57 | 0.24 |
|  | 30d | 61.97 | 28.73 | 26.77 | 0.87 | 0.84 | 3.01 | 0.85 | 0.66 | 0.18 |

- No detection
