# Supplementary material for: Personalized neoantigen vaccine prevents postoperative recurrence in hepatocellular carcinoma patients with vascular invasion
Source: Mol Cancer. 2021 Dec 13;20:164. doi: 10.1186/s12943-021-01467-8 (PMC8667400; doi:10.1186/s12943-021-01467-8)
Supplement: Supplementary file 15 — Additional file 15: Supplementary Table S6. New neoantigen mutations identified in recurrent tumor. [file 12943_2021_1467_MOESM15_ESM.docx]

**Supplementary Table S6. New neoantigen mutations in recurrent tumor**

| **Chr_Pos** | **ref** | **alt** | **gene** | **Primary tumor_freq** | **Recurrent tumor_freq** | **corresponding HLA types** |
| --- | --- | --- | --- | --- | --- | --- |
| chr1_40705080 | A | T | RLF | 0 | 0.165562914 | HLA-A*11:01,HLA-DRB1*08:03 |
| chr1_54291524 | T | C | NDC1 | 0 | 0.347368421 | HLA-A*24:02,HLA-DRB1*08:03 |
| chr2_29016794 | T | G | PPP1CB | 0 | 0.169014085 | HLA-A*11:01,HLA-DRB1*08:03 |
| chr2_223289376 | C | A | SGPP2 | 0 | 0.157024793 | HLA-A*24:02 |
| chr3_49847264 | T | A | UBA7 | 0 | 0.174157303 | HLA-A*24:02 |
| chr8_19297437 | A | T | CSGALNACT1 | 0 | 0.253731343 | HLA-A*11:01 |
| chr15_72640064 | T | A | HEXA | 0 | 0.185185185 | HLA-A*24:02 |
| chr22_30811952 | G | A | SEC14L2 | 0 | 0.175824176 | HLA-A*11:01 |
| chrX_70347191 | G | C | MED12 | 0 | 0.344827586 | HLA-A*11:01 |
